# Supplementary figures and images for: Legionella effector LpPIP recruits protein phosphatase 1 to the mitochondria to induce dephosphorylation of outer membrane proteins
Source: PLoS Biol. 2025 Jul 23;23(7):e3003261. doi: 10.1371/journal.pbio.3003261 (PMC12313075; doi:10.1371/journal.pbio.3003261)

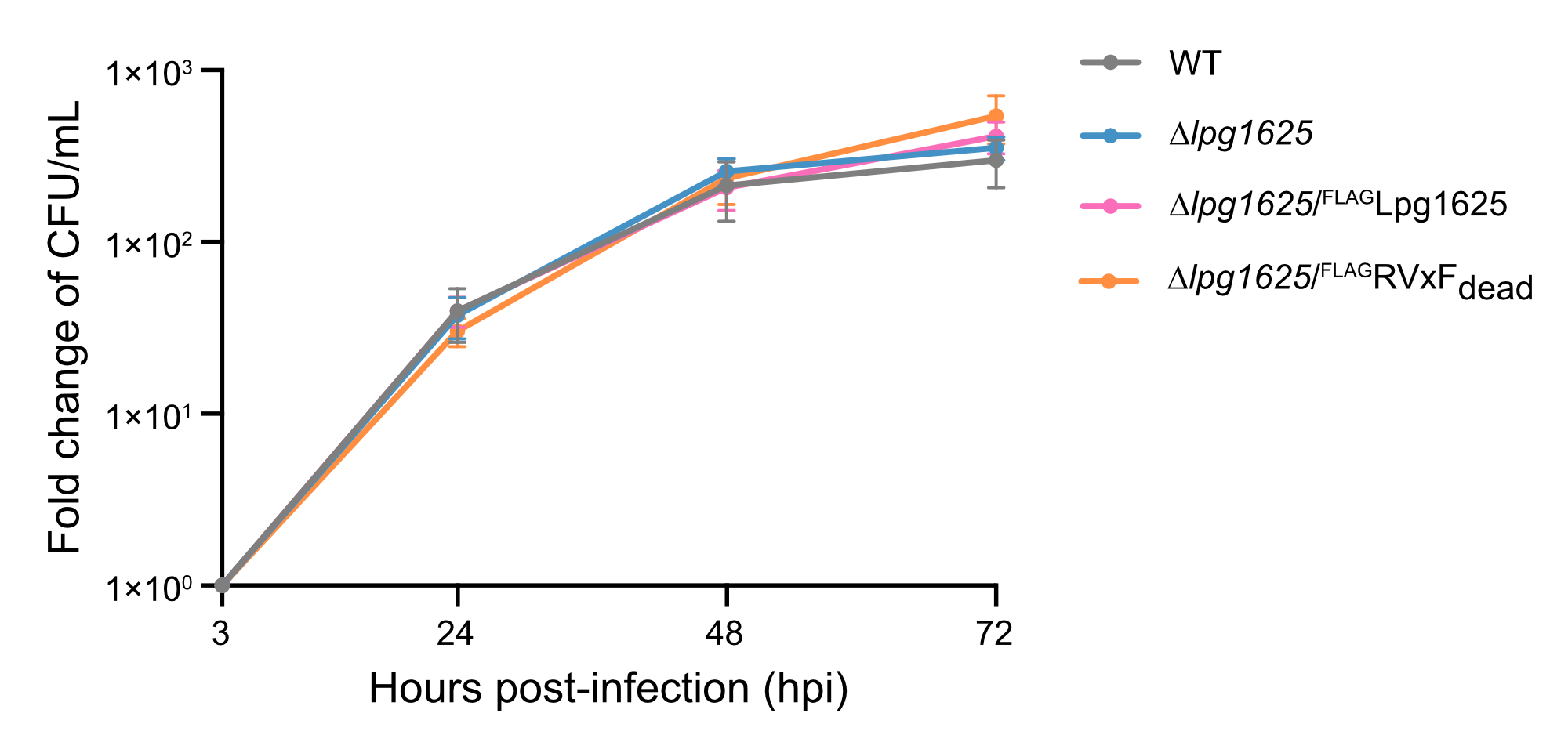

Supplement: S1 Fig — Differentiated THP-1 cells were infected with L. pneumophila JR32 (wild-type), Δlpg1625, Δlpg1625 complemented with FLAGLpg1625 or FLAGRVxFdead (FLAGLpg1625K17A/V19A, mutant that loses interaction with PP1) with multiplicity of infection (MOI) of 5 (n = 5 independent experiments). The mean ± SEM for fold change of the colony-forming units (CFU/mL) of the different L. pneumophila strains from THP-1 cells infected over three days against 3 hpi were plotted. Two-way ANOVA was used for statistical analysis. Corresponding raw data are provided in S2 Table. (TIFF) [file pbio.3003261.s001.tiff]

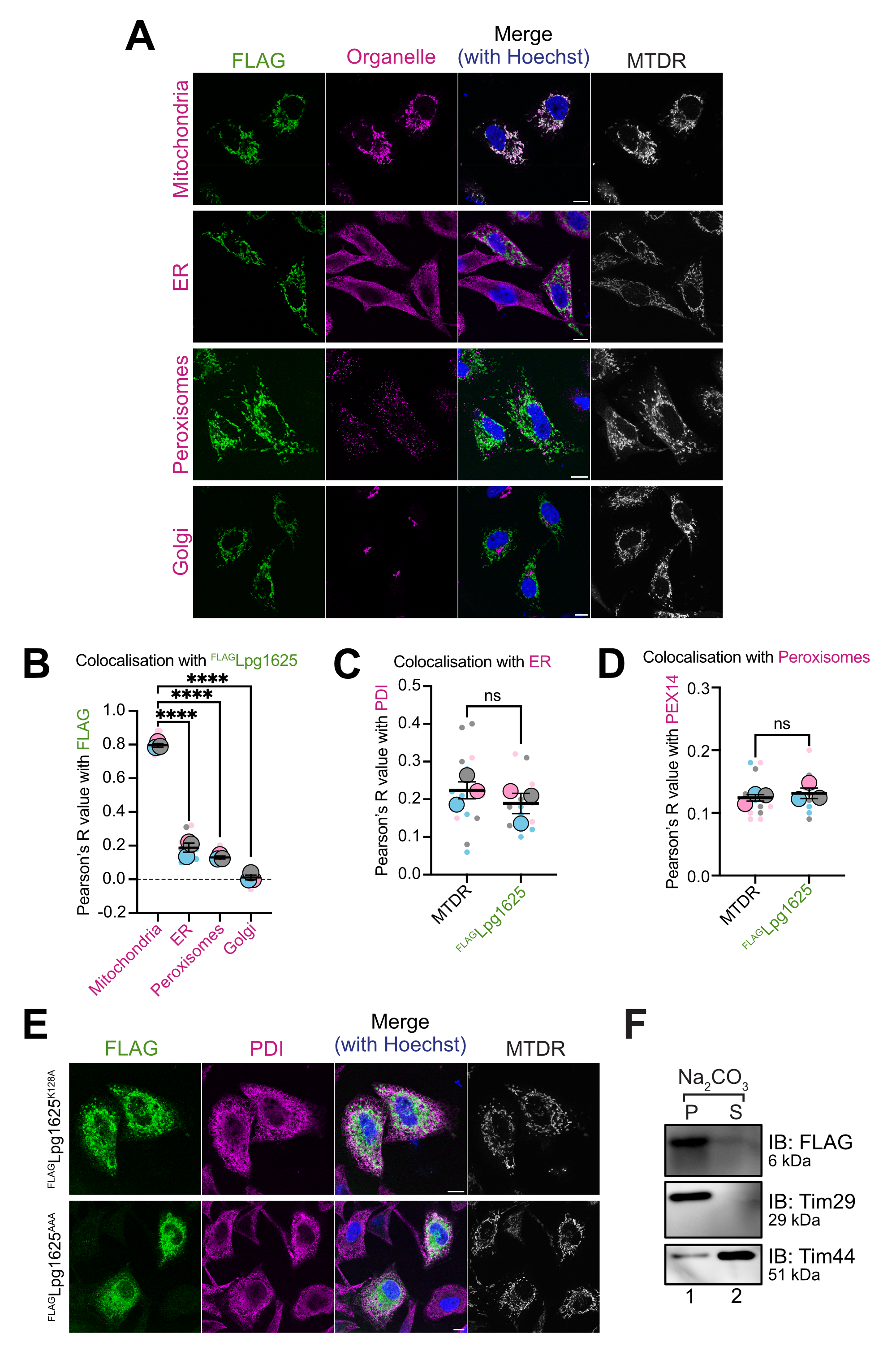

Supplement: S2 Fig — (A) HeLa cells transiently transfected with FLAGLpg1625 were stained with 100 nM MitoTracker Deep Red FM (mitochondria; MTDR) and immunostained with antibodies against FLAG (green) and different organellar markers (magenta): NDUFAF2 (mitochondria), PDI (ER), PEX14 (peroxisomes), and GM130 (Golgi). Nuclei were stained with Hoechst 33258 (blue). Scale bar represents 10 μm. (B) The colocalization between FLAGLpg1625 and different organellar markers was quantified using Pearson correlation coefficient (r) (n = 3 independent experiments, 5 cells each). Data represent mean ± SEM of the three experiments, with representative images presented in S2A Fig. Ordinary one-way ANOVA was performed on the means, comparing them to mitochondrial colocalization. Colocalization of FLAGLpg1625 with all organellar markers was significantly lower than that with mitochondria, with a p-value < 0.0001. Corresponding raw data are available in S1 Data. (C) Colocalization between PDI and MTDR/FLAGLpg1625 was quantified using Pearson correlation coefficient (r) (n = 3 independent experiments, 5 cells each). Data represents mean ± SEM of the three experiments, with representative images presented in S2A Fig. Unpaired t test was performed on the means, showing no significant difference. Corresponding raw data are available in S1 Data. (D) The colocalization between PEX14 and MTDR/FLAGLpg1625 was quantified using Pearson correlation coefficient (r) (n = 3 independent experiments, 5 cells each). Data represents mean ± SEM of the three experiments, with representative images presented in S2A Fig. Unpaired t test was performed on the means, showing no significant difference. Corresponding raw data are available in S1 Data. (E) HeLa cells transiently transfected with FLAGLpg1625K128A and FLAGLpg1625AAA (K97A/R100A/K128A) were stained with 100 nM MitoTracker Deep Red FM (mitochondria; MTDR) and immunostained with antibodies against FLAG (green) and PDI (ER; magenta). Nuclei were stained with Hoechst 332 [file pbio.3003261.s002.tiff]

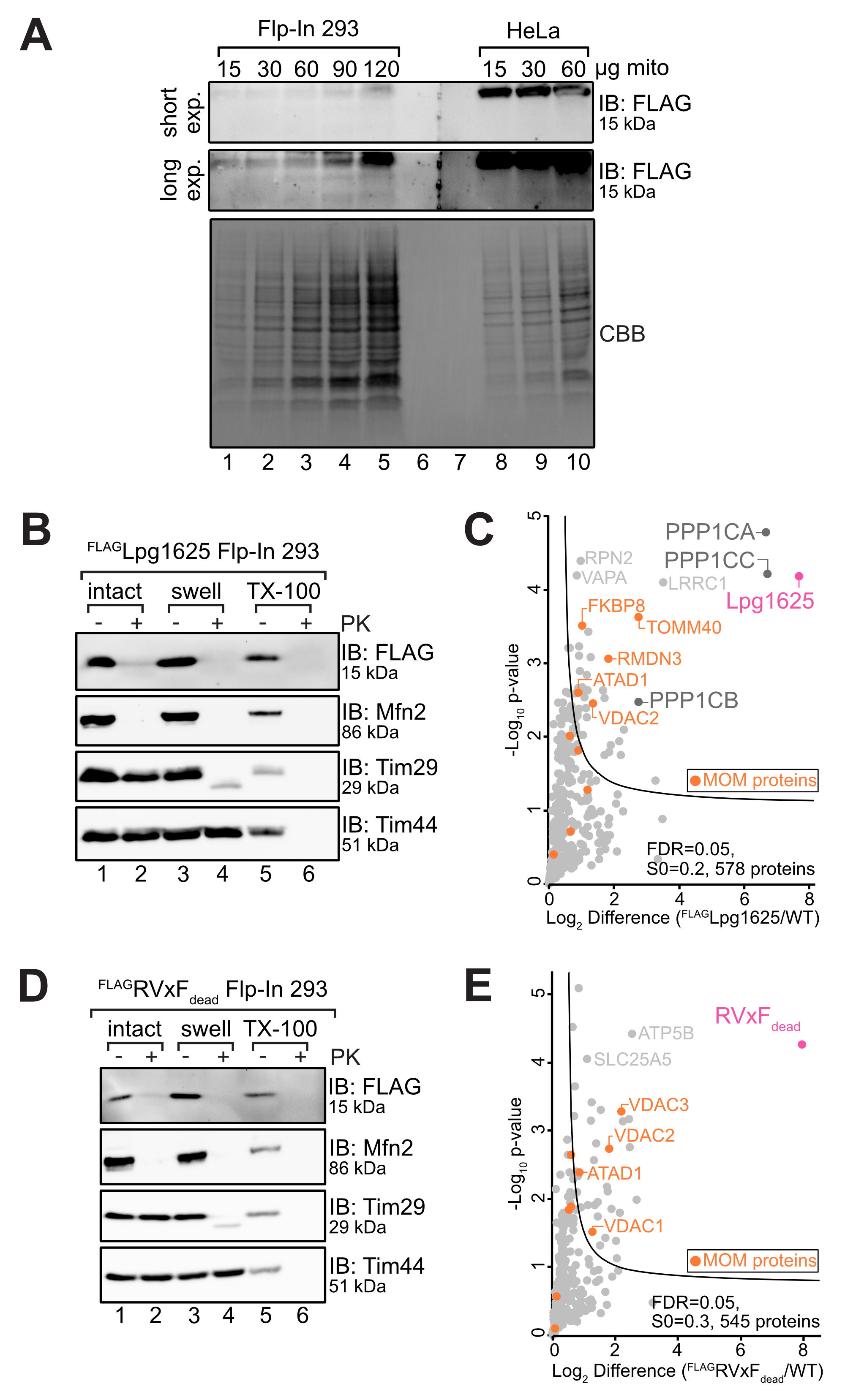

Supplement: S3 Fig — (A) Titration of mitochondria isolated from tetracycline-induced Flp-In T-REx 293 stable cells expressing FLAGLpg1625 and from HeLa cells transiently transfected with FLAGLpg1625. Coomassie brilliant blue staining was used as a loading control. Corresponding raw images are available in S1 Raw Images, (B) Mitochondria sub-fractionation with mitochondria isolated from Flp-In T-REx 293 stable cell lines of FLAGLpg1625. Isolated mitochondria were either left intact (lane 1 and 2), subjected to hypotonic swelling (lane 3 and 4), or solubilized with 0.5% Triton X-100 (lane 5 and 6). These samples were either left untreated or treated with 50 µg/mL Proteinase K (PK) and analyzed with SDS-PAGE and immunoblotting with the indicated antibodies. Corresponding raw images are available in S1 Raw Images. (C) Volcano plot showing proteins enriched in FLAG co-immunoprecipitation from mitochondria isolated from Flp-In T-REx 293 stable cell lines expressing FLAGLpg1625 compared to wild-type Flp-In T-REx 293. The Log2 fold change of mean LFQ intensity is plotted against −Log10 p-value (n = 3 technical replicates). The curve indicates significantly enriched proteins (FDR = 0.05, s0 = 0.2). Mitochondrial outer membrane proteins are labeled with the annotation from MitoCarta3.0. Corresponding data are available in S4 Table. (D) Mitochondria sub-fractionation on mitochondria isolated from Flp-In T-REx 293 stable cell line of FLAGRVxFdead was performed as described in S3B Fig. Corresponding raw images are available in S1 Raw Images. (E) Volcano plot showing proteins enriched in FLAG co-immunoprecipitation samples of mitochondria isolated from Flp-In T-REx 293 stable cell lines of FLAGRVxFdead compared to wild-type Flp-In T-REx 293. The Log2 fold change of mean LFQ intensity is plotted against −Log10 p-value (n = 3 technical replicates). The curve indicates significantly enriched proteins (FDR = 0.05, s0 = 0.3). Mitochondrial outer membrane proteins are labeled with the annotation from Mito [file pbio.3003261.s003.tiff]

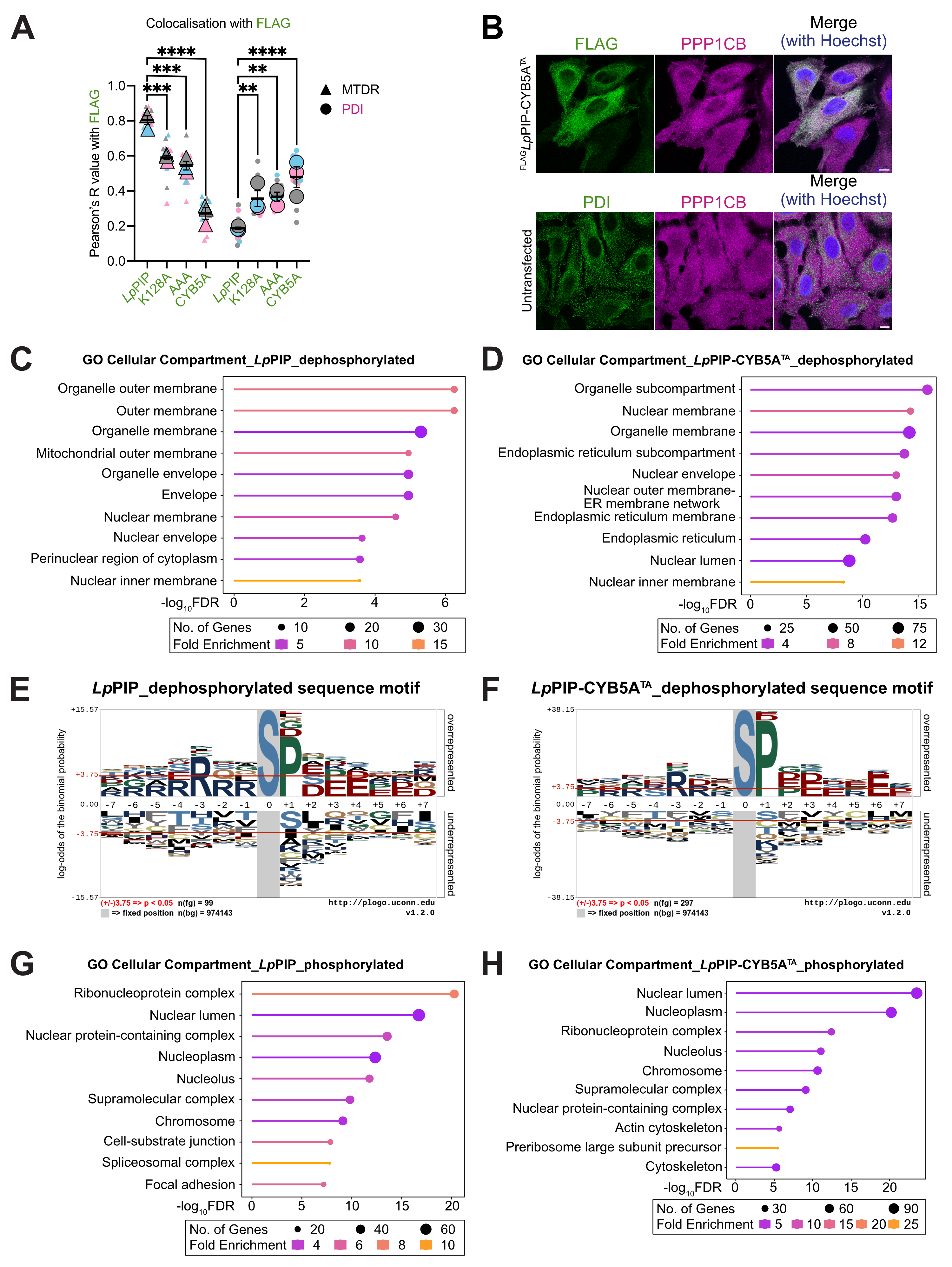

Supplement: S4 Fig — (A) Colocalization between FLAGLpPIP/FLAGLpPIPK128A/FLAGLpPIPAAA/FLAGLpPIP-CYB5ATA and mitochondria/ER was quantified using Pearson correlation coefficient (r) (n = 3 independent experiments, 5 cells each). Data represent mean ± SEM of the three experiments, with representative images presented in Figs 5A and S2E. Ordinary two-way ANOVA was performed on the means, comparing them to FLAGLpPIP within each group (MTDR or PDI). Colocalization of FLAGLpPIP with mitochondria (MTDR) was significantly higher than all the other variants, while colocalization of FLAGLpPIP with ER (PDI) was significantly lower than all the other variants. Corresponding raw data are available in S1 Data. (B) Representative images for Fig 5B. Untransfected HeLa cells and cells transiently transfected with FLAGLpPIP-CYB5ATA were immunostained with antibodies against FLAG (green)/PDI (ER; green) and PPP1CB (magenta). Nuclei were stained with Hoechst 33258 (blue). Scale bar represents 10 μm. (C) All proteins with phosphopeptides significantly reduced in abundance in HeLa cells transfected with FLAGLpPIP compared to empty vector pCDNA5 (Fig 5E) were used for gene ontology (GO) cellular compartment enrichment analysis with ShinyGO 0.80 [57]. The top 10 GO biological process terms are shown. Corresponding data are available in S2 Data. (D) Proteins with phosphopeptides significantly reduced in abundance in HeLa cells transfected with FLAGLpPIP-CYB5ATA compared to empty vector pCDNA5 (Fig 5F) were used for gene ontology (GO) cellular compartment enrichment analysis with ShinyGO 0.80 [57]. The top 10 GO biological process terms were shown. Corresponding data are available in S2 Data. (E) pLogo [56] sequence motif visualization of seven residues flanking 115 pSer/pThr sites found dephosphorylated in the presence of FLAGLpPIP, as shown in Fig 5E. Arginine (R) (log-odds = 7.182, p-value = 1.843 e−5) and proline (P) (log-odds = 10.779, p-value = 4.653 e−9) at position −3 and +1 relative to pSer/pThr respect [file pbio.3003261.s004.tiff]
