# Supplementary material for: Legionella effector LpPIP recruits protein phosphatase 1 to the mitochondria to induce dephosphorylation of outer membrane proteins
Source: PLoS Biol. 2025 Jul 23;23(7):e3003261. doi: 10.1371/journal.pbio.3003261 (PMC12313075; doi:10.1371/journal.pbio.3003261)
Supplement: S1 Text — (DOCX) [file pbio.3003261.s012.docx]

|  | **Locus name** | **Gene name** | **Uniprot accession** | **Reference** |
| --- | --- | --- | --- | --- |
| 1 | Lpg0008 | ravA | Q5ZZK2 | [1] |
| 2 | Lpg0012 | CegC1 | Q5ZZJ8 | [2] |
| 3 | Lpg0021 | N/A | Q5ZZI9 | [3] |
| 4 | Lpg0030 | ravB | Q5ZZI0 | [1] |
| 5 | Lpg0038 | LegA10 | Q5ZZH2 | [4] |
| 6 | Lpg0041 | N/A | Q5ZZG9 | [2] |
| 7 | Lpg0045 | N/A | Q5ZZG5 | [5] |
| 8 | Lpg0046 | N/A | Q5ZZG4 | [3] |
| 9 | Lpg0059 | Ceg2 | Q5ZZF1 | [6] |
| 10 | Lpg0080 | Ceg3 | Q5ZZD1 | [6] |
| 11 | Lpg0081 | N/A | Q5ZZD0 | [5] |
| 12 | Lpg0090 | Lem1 | Q5ZZC1 | [6] |
| 13 | Lpg0096 | Ceg4 | Q5ZZB5 | [6] |
| 14 | Lpg0103 | VipF | Q5ZZA8 | [7] |
| 15 | Lpg0126 | CegC2 | Q5ZZ85 | [2] |
| 16 | Lpg0130 | N/A | Q5ZZ81 | [3] |
| 17 | Lpg0160 | ravD | Q5ZZ51 | [1] |
| 18 | Lpg0170 | ravC | Q5ZZ41 | [1] |
| 19 | Lpg0171 | LegU1 | Q5ZZ40 | [4] |
| 20 | Lpg0172 | N/A | Q5ZZ39 | [3] |
| 21 | Lpg0181 | Lart1 | Q5ZZ30 | [3] |
| 22 | Lpg0191 | Ceg5 | Q5ZZ20 | [6] |
| 23 | Lpg0195 | ravE | Q5ZZ16 | [1] |
| 24 | Lpg0196 | ravF | Q5ZZ15 | [1] |
| 25 | Lpg0210 | ravG | Q5ZZ01 | [1] |
| 26 | Lpg0227 | Ceg7 | Q5ZYY4 | [2] |
| 27 | Lpg0234 | SidE | Q5ZYX7 | [8] |
| 28 | Lpg0240 | Ceg8 | Q5ZYX1 | [6] |
| 29 | Lpg0246 | Ceg9 | Q5ZYW5 | [9] |
| 30 | Lpg0260 | N/A | Q5ZYV1 | [3] |
| 31 | Lpg0276 | LegG2 | Q5ZYT5 | [4] |
| 32 | Lpg0284 | Ceg10 | Q5ZYS7 | [10] |
| 33 | Lpg0285 | Lem2 | Q5ZYS6 | [6] |
| 34 | Lpg0294 | dotY | Q5ZYR7 | [6] |
| 35 | Lpg0364 | N/A | Q5ZYK8 | [3] |
| 36 | Lpg0365 | N/A | Q5ZYK7 | [5] |
| 37 | Lpg0375 | N/A | Q5ZYJ7 | [3] |
| 38 | Lpg0376 | sdhA | Q5ZYJ6 | [8] |
| 39 | Lpg0390 | VipA | Q5ZYI2 | [7] |
| 40 | Lpg0401 | Ceg11 | Q5ZYH1 | [1] |
| 41 | Lpg0402 | LegA9 | Q5ZYH0 | [4] |
| 42 | Lpg0403 | LegA7 | Q5ZYG9 | [11] |
| 43 | Lpg0405 | N/A | Q5ZYG7 | [3] |
| 44 | Lpg0422 | LegY | Q5ZYF0 | [4] |
| 45 | Lpg0436 | LegA11 | Q5ZYD6 | [4] |
| 46 | Lpg0437 | Ceg14 | Q5ZYD5 | [6] |
| 47 | Lpg0439 | Ceg15 | Q5ZYD3 | [1] |
| 48 | Lpg0483 | LegA12 | Q5ZY89 | [4] |
| 49 | Lpg0515 | LegD2 | Q5ZY57 | [4] |
| 50 | Lpg0518 | N/A | Q5ZY54 | [5] |
| 51 | Lpg0519 | Ceg17 | Q5ZY53 | [6] |
| 52 | Lpg0621 | SidA | Q5ZXW0 | [8] |
| 53 | Lpg0634 | N/A | Q5ZXU7 | [5] |
| 54 | Lpg0642 | WipB | Q5ZXT9 | [12] |
| 55 | Lpg0695 | LegA8 | Q5ZXN6 | [4] |
| 56 | Lpg0696 | Lem3 | Q5ZXN5 | [6] |
| 57 | Lpg0716 | N/A | Q5ZXL5 | [3] |
| 58 | Lpg0733 | ravH | Q5ZXJ8 | [1] |
| 59 | Lpg0796 | N/A | Q5ZXD5 | [3] |
| 60 | Lpg0898 | Ceg18 | Q5ZX35 | [2] |
| 61 | Lpg0926 | ravI | Q5ZX07 | [1] |
| 62 | Lpg0940 | LidA | Q5ZWZ3 | [13] |
| 63 | Lpg0944 | ravJ | Q5ZWY9 | [1] |
| 64 | Lpg0945 | LegL1 | Q5ZWY8 | [4] |
| 65 | Lpg0963 | N/A | Q5ZWX1 | [5] |
| 66 | Lpg0967 | N/A | Q5ZWW7 | [3] |
| 67 | Lpg0968 | SidK | Q5ZWW6 | [14] |
| 68 | Lpg0969 | ravK | Q5ZWW5 | [1] |
| 69 | Lpg1083 | N/A | Q5ZWK2 | [3] |
| 70 | Lpg1101 | Lem4 | Q5ZWI4 | [6] |
| 71 | Lpg1106 | N/A | Q5ZWH9 | [3] |
| 72 | Lpg1108 | ravL | Q5ZWH7 | [1] |
| 73 | Lpg1109 | ravM | Q5ZWH6 | [1] |
| 74 | Lpg1110 | Lem5 | Q5ZWH5 | [6] |
| 75 | Lpg1111 | ravN | Q5ZWH4 | [1] |
| 76 | Lpg1120 | Lem6 | Q5ZWG5 | [6] |
| 77 | Lpg1121 | Ceg19 | Q5ZWG4 | [6] |
| 78 | Lpg1124 | N/A | Q5ZWG1 | [3] |
| 79 | Lpg1129 | ravO | Q5ZWF6 | [1] |
| 80 | Lpg1137 | N/A | Q5ZWE8 | [3] |
| 81 | Lpg1144 | CegC3 | Q5ZWE1 | [2] |
| 82 | Lpg1145 | Lem7 | Q5ZWE0 | [6] |
| 83 | Lpg1147 | N/A | Q5ZWD8 | [3] |
| 84 | Lpg1148 | N/A | Q5ZWD7 | [5] |
| 85 | Lpg1152 | ravP | Q5ZWD3 | [1] |
| 86 | Lpg1154 | ravQ | Q5ZWD1 | [1] |
| 87 | Lpg1158 | N/A | Q5ZWC7 | [5] |
| 88 | Lpg1166 | ravR | Q5ZWB9 | [1] |
| 89 | Lpg1171 | N/A | Q5ZWB4 | [3] |
| 90 | Lpg1183 | ravS | Q5ZWA2 | [1] |
| 91 | Lpg1227 | VpdB | Q5ZW60 | [15] |
| 92 | Lpg1273 | N/A | Q5ZW15 | [5] |
| 93 | Lpg1290 | Lem8 | Q5ZVZ8 | [6] |
| 94 | Lpg1312 | legC1 | Q5ZVX6 | [4] |
| 95 | Lpg1316 | ravT | Q5ZVX2 | [1] |
| 96 | Lpg1317 | ravW | Q5ZVX1 | [1] |
| 97 | Lpg1328 | LegT | Q5ZVW1 | [4] |
| 98 | Lpg1355 | SidG | Q5ZVT5 | [8] |
| 99 | Lpg1426 | VpdC | Q5ZVL4 | [15] |
| 100 | Lpg1449 | N/A | Q5ZVJ1 | [3] |
| 101 | Lpg1453 | N/A | Q5ZVI7 | [3] |
| 102 | Lpg1483 | LegK1 | Q5ZVF7 | [4] |
| 103 | Lpg1484 | N/A | Q5ZVF6 | [3] |
| 104 | Lpg1488 | LegC5 | Q5ZVF2 | [4] |
| 105 | Lpg1489 | ravX | Q5ZVF1 | [1] |
| 106 | Lpg1491 | Lem9 | Q5ZVE9 | [6] |
| 107 | Lpg1496 | Lem10 | Q5ZVE4 | [6] |
| 108 | Lpg1551 | ravY | Q5ZV89 | [1] |
| 109 | Lpg1578 | N/A | Q5ZV62 | [3] |
| 110 | Lpg1588 | LegC6 | Q5ZV52 | [4] |
| 111 | Lpg1598 | Lem11 | Q5ZV42 | [6] |
| 112 | Lpg1602 | LegL2 | Q5ZV40 | [4] |
| 113 | Lpg1621 | Ceg23 | Q5ZV21 | [10] |
| 114 | Lpg1625 | Lem12 | Q5ZV17 | [6] |
| 115 | Lpg1639 | N/A | Q5ZV03 | [3] |
| 116 | Lpg1642 | SidB | Q5ZV00 | [8] |
| 117 | Lpg1654 | N/A | Q5ZUY8 | [3] |
| 118 | Lpg1660 | LegL3 | Q5ZUY2 | [4] |
| 119 | Lpg1661 | N/A | Q5ZUY1 | [3] |
| 120 | Lpg1666 | N/A | Q5ZUX6 | [3] |
| 121 | Lpg1667 | N/A | Q5ZUX5 | [3] |
| 122 | Lpg1670 | N/A | Q5ZUX2 | [3] |
| 123 | Lpg1683 | ravZ | Q5ZUV9 | [1] |
| 124 | Lpg1684 | N/A | Q5ZUV8 | [3] |
| 125 | Lpg1685 | N/A | Q5ZUV7 | [3] |
| 126 | Lpg1687 | mavA | Q5ZUV5 | [1] |
| 127 | Lpg1689 | N/A | P37033 | [5] |
| 128 | Lpg1692 | N/A | Q5ZUV0 | [3] |
| 129 | Lpg1701 | LegC3 | Q5ZUU1 | [4] |
| 130 | Lpg1702 | PpeB | Q5ZUU0 | [16] |
| 131 | Lpg1716 | N/A | Q5ZUS6 | [3] |
| 132 | Lpg1717 | N/A | Q5ZUS5 | [5] |
| 133 | Lpg1718 | LegAS4 | Q5ZUS4 | [4] |
| 134 | Lpg1751 | N/A | Q5ZUP2 | [5] |
| 135 | Lpg1752 | N/A | Q5ZUP1 | [1] |
| 136 | Lpg1776 | N/A | Q5ZUL7 | [3] |
| 137 | Lpg1797 | rvfA | Q5ZUK1 | [1] |
| 138 | Lpg1798 | marB | Q5ZUK0 | [1] |
| 139 | Lpg1803 | N/A | Q5ZUJ5 | [3] |
| 140 | Lpg1851 | Lem14 | Q5ZUE7 | [6] |
| 141 | Lpg1884 | YlfB | Q5ZUC3 | [17] |
| 142 | Lpg1888 | N/A | Q5ZUB9 | [3] |
| 143 | Lpg1890 | LegLC8 | Q5ZUB7 | [4] |
| 144 | Lpg1907 | N/A | Q5ZUA0 | [3] |
| 145 | Lpg1924 | N/A | Q5ZU83 | [3] |
| 146 | Lpg1933 | Lem15 | Q5ZU75 | [6] |
| 147 | Lpg1947 | Lem16 | Q5ZU61 | [6] |
| 148 | Lpg1948 | LegLC4 | Q5ZU60 | [4] |
| 149 | Lpg1949 | Lem17 | Q5ZU59 | [6] |
| 150 | Lpg1950 | RalF | Q5ZU58 | [18] |
| 151 | Lpg1953 | LegC4 | Q5ZU55 | [4] |
| 152 | Lpg1958 | LegL5 | Q5ZU50 | [4] |
| 153 | Lpg1959 | N/A | Q5ZU49 | [3] |
| 154 | Lpg1960 | LirA | Q5ZU48 | [19] |
| 155 | Lpg1962 | LirB | Q5ZU46 | [19] |
| 156 | Lpg1963 | LirC | Q5ZU45 | [19] |
| 157 | Lpg1964 | LirD | Q5ZU44 | [19] |
| 158 | Lpg1965 | LirE | Q5ZU43 | [19] |
| 159 | Lpg1966 | LirF | Q5ZU42 | [19] |
| 160 | Lpg1969 | PieE | Q5ZU39 | [16] |
| 161 | Lpg1972 | PieF | Q5ZU36 | [16] |
| 162 | Lpg1976 | LegG1 | Q5ZU32 | [4] |
| 163 | Lpg1978 | SetA | Q5ZU30 | [9] |
| 164 | Lpg1986 | N/A | Q5ZU22 | [3] |
| 165 | Lpg2050 | N/A | Q5ZTV8 | [3] |
| 166 | Lpg2131 | LegA6 | Q5ZTN0 | [4] |
| 167 | Lpg2137 | LegK2 | Q5ZTM4 | [4] |
| 168 | Lpg2144 | LegAU13 | Q5ZTL7 | [4] |
| 169 | Lpg2147 | mavC | Q5ZTL4 | [1] |
| 170 | Lpg2148 | N/A | Q5ZTL3 | [3] |
| 171 | Lpg2149 | N/A | Q5ZTL2 | [3] |
| 172 | Lpg2153 | SdeC | Q5ZTK8 | [8] |
| 173 | Lpg2155 | SidJ | Q5ZTK6 | [20] |
| 174 | Lpg2156 | SdeB | Q5ZTK5 | [19] |
| 175 | Lpg2157 | SdeA | Q5ZTK4 | [8] |
| 176 | Lpg2166 | Lem19 | Q5ZTJ5 | [6] |
| 177 | Lpg2176 | LegS2 | Q5ZTI6 | [4] |
| 178 | Lpg2199 | mavD | Q5ZTG3 | [1] |
| 179 | Lpg2200 | CegC4 | Q5ZTG2 | [2] |
| 180 | Lpg2215 | LegA2 | Q5ZTE7 | [4] |
| 181 | Lpg2216 | Lem20 | Q5ZTE6 | [6] |
| 182 | Lpg2223 | N/A | Q5ZTD9 | [3] |
| 183 | Lpg2224 | PpgA | Q5ZTD8 | [16] |
| 184 | Lpg2239 | N/A | Q5ZTC3 | [3] |
| 185 | Lpg2248 | Lem21 | Q5ZTB4 | [6] |
| 186 | Lpg2271 | N/A | Q5ZT91 | [3] |
| 187 | Lpg2298 | YlfA | Q5ZT67 | [17] |
| 188 | Lpg2300 | LegA3 | Q5ZT65 | [4] |
| 189 | Lpg2311 | Ceg28 | Q5ZT54 | [1] |
| 190 | Lpg2322 | LegA5 | Q5ZT43 | [4] |
| 191 | Lpg2327 | N/A | Q5ZT38 | [5] |
| 192 | Lpg2328 | Lem22 | Q5ZT37 | [6] |
| 193 | Lpg2344 | mavE | Q5ZT21 | [1] |
| 194 | Lpg2351 | mavF | Q5ZT14 | [1] |
| 195 | Lpg2359 | N/A | Q5ZT06 | [3] |
| 196 | Lpg2370 | N/A | Q5ZSZ6 | [3] |
| 197 | Lpg2372 | N/A | Q5ZSZ4 | [3] |
| 198 | Lpg2382 | N/A | Q5ZSY4 | [3] |
| 199 | Lpg2391 | sdbC | Q5ZSX5 | [1] |
| 200 | Lpg2392 | LegL6 | Q5ZSX4 | [4] |
| 201 | Lpg2400 | LegL7 | Q5ZSW6 | [4] |
| 202 | Lpg2406 | Lem23 | Q5ZSW0 | [6] |
| 203 | Lpg2407 | N/A | Q5ZSV9 | [5] |
| 204 | Lpg2409 | Ceg29 | Q5ZSV7 | [10] |
| 205 | Lpg2410 | VpdA | Q5ZSV6 | [15] |
| 206 | Lpg2411 | Lem24 | Q5ZSV5 | [6] |
| 207 | Lpg2416 | LegA1 | Q5ZSV0 | [4] |
| 208 | Lpg2420 | N/A | Q5ZSU6 | [1] |
| 209 | Lpg2422 | Lem25 | Q5ZSU4 | [6] |
| 210 | Lpg2424 | mavG | Q5ZSU2 | [1] |
| 211 | Lpg2425 | mavH | Q5ZSU1 | [1] |
| 212 | Lpg2433 | Ceg30 | Q5ZST3 | [6] |
| 213 | Lpg2434 | N/A | Q5ZST2 | [3] |
| 214 | Lpg2443 | N/A | Q5ZSS3 | [3] |
| 215 | Lpg2444 | mavI | Q5ZSS2 | [1] |
| 216 | Lpg2452 | LegA14 | Q5ZSR5 | [4] |
| 217 | Lpg2456 | LegA15 | Q5ZSR1 | [4] |
| 218 | Lpg2461 | N/A | Q5ZSQ6 | [3] |
| 219 | Lpg2464 | SidM | Q5ZSQ3 | [21] |
| 220 | Lpg2465 | SidD | Q5ZSQ2 | [8] |
| 221 | Lpg2490 | LepB | Q5ZSM7 | [22] |
| 222 | Lpg2498 | mavJ | Q5ZSL9 | [1] |
| 223 | Lpg2504 | SidI | Q5ZSL3 | [23] |
| 224 | Lpg2505 | N/A | Q5ZSL2 | [3] |
| 225 | Lpg2508 | SdjA | Q5ZSK9 | [20] |
| 226 | Lpg2509 | SdeD | Q5ZSK8 | [8] |
| 227 | Lpg2510 | SdcA | Q5ZSK7 | [8] |
| 228 | Lpg2511 | SidC | Q5ZSK6 | [8] |
| 229 | Lpg2523 | Lem26 | Q5ZSJ4 | [6] |
| 230 | Lpg2525 | mavK | Q5ZSJ2 | [1] |
| 231 | Lpg2526 | mavL | Q5ZSJ1 | [1] |
| 232 | Lpg2527 | N/A | Q5ZSJ0 | [5] |
| 233 | Lpg2529 | Lem27 | Q5ZSI8 | [6] |
| 234 | Lpg2538 | N/A | Q5ZSH9 | [3] |
| 235 | Lpg2539 | N/A | Q5ZSH8 | [3] |
| 236 | Lpg2541 | N/A | Q5ZSH6 | [24] |
| 237 | Lpg2546 | N/A | Q5ZSH1 | [3] |
| 238 | Lpg2552 | N/A | Q5ZSG5 | [1] |
| 239 | Lpg2555 | N/A | Q5ZSG2 | [3] |
| 240 | Lpg2556 | LegK3 | Q5ZSG1 | [4] |
| 241 | Lpg2577 | mavM | Q5ZSE2 | [1] |
| 242 | Lpg2584 | SidF | Q5ZSD5 | [8] |
| 243 | Lpg2588 | LegS1 | Q5ZSD1 | [4] |
| 244 | Lpg2591 | Ceg33 | Q5ZSC8 | [2] |
| 245 | Lpg2603 | Lem28 | Q5ZSB6 | [6] |
| 246 | Lpg2628 | N/A | Q5ZS91 | [3] |
| 247 | Lpg2637 | N/A | Q5ZS82 | [3] |
| 248 | Lpg2638 | mavV | Q5ZS81 | [1] |
| 249 | Lpg2692 | N/A | Q5ZS27 | [3] |
| 250 | Lpg2694 | LegD1 | Q5ZS25 | [4] |
| 251 | Lpg2718 | WipA | Q5ZS02 | [12] |
| 252 | Lpg2720 | LegN | Q5ZS00 | [4] |
| 253 | Lpg2744 | N/A | Q5ZRX7 | [5] |
| 254 | Lpg2745 | N/A | Q5ZRX6 | [3] |
| 255 | Lpg2793 | LepA | Q5ZRT5 | [22] |
| 256 | Lpg2804 | Lem29 | Q5ZRS6 | [6] |
| 257 | Lpg2815 | mavN | Q5ZRR5 | [1] |
| 258 | Lpg2826 | Ceg34 | Q5ZRQ4 | [6] |
| 259 | Lpg2828 | N/A | Q5ZRQ2 | [3] |
| 260 | Lpg2829 | SidH | Q5ZRQ1 | [8] |
| 261 | Lpg2830 | LegU2 | Q5ZRQ0 | [4] |
| 262 | Lpg2831 | VipD | Q5ZRP9 | [7] |
| 263 | Lpg2832 | N/A | Q5ZRP8 | [3] |
| 264 | Lpg2844 | N/A | Q5ZRN6 | [3] |
| 265 | Lpg2862 | LegC8 | Q5ZRL9 | [4] |
| 266 | Lpg2874 | N/A | Q5ZRK7 | [1] |
| 267 | Lpg2879 | mavO | Q5ZRK2 | [1] |
| 268 | Lpg2884 | mavP | Q5ZRJ7 | [1] |
| 269 | Lpg2885 | N/A | Q5ZRJ6 | [3] |
| 270 | Lpg2888 | N/A | Q5ZRJ3 | [3] |
| 271 | Lpg2912 | N/A | Q5ZRH0 | [3] |
| 272 | Lpg2936 | N/A | Q5ZRE6 | [3] |
| 273 | Lpg2975 | mavQ | Q5ZRA8 | [1] |
| 274 | Lpg2999 | LegP | Q5ZR84 | [4] |
| 275 | Lpg3000 | N/A | Q5ZR83 | [3] |
| 276 | Lpg0140 | CetLp1 | Q5ZZ71 | [25] |
| 277 | Lpg0393 | N/A | Q5ZYH9 | [25] |
| 278 | Lpg1663 | N/A | Q5ZUX9 | [25] |
| 279 | Lpg1822 | N/A | Q5ZUH6 | [25] |
| 280 | Lpg2244 | N/A | Q5ZTB8 | [25] |
| 281 | Lpg2283 | N/A | Q5ZT79 | [25] |
| 282 | Lpg2806 | N/A | Q5ZRS4 | [25] |
| 283 | Lpg0107 | N/A | Q5ZZA4 | [26] |
| 284 | Lpg0135 | sdhB | Q5ZZ76 | [26] |
| 285 | Lpg0208 | pkn5 | Q5ZZ03 | [26] |
| 286 | Lpg0209 | N/A | Q5ZZ02 | [26] |
| 287 | Lpg0254 | N/A | Q5ZYV7 | [26] |
| 288 | Lpg0275 | sdbA | Q5ZYT6 | [26] |
| 289 | Lpg0770 | N/A | Q5ZXG1 | [26] |
| 290 | Lpg0921 | N/A | Q5ZX12 | [26] |
| 291 | Lpg1368 | Lgt1 | Q5ZVS2 | [26] |
| 292 | Lpg1408 | licA | Q5ZVN2 | [26] |
| 293 | Lpg1836 | N/A | Q5ZUG2 | [26] |
| 294 | Lpg1961 | N/A | Q5ZU47 | [26] |
| 295 | Lpg2160 | N/A | Q5ZTK1 | [26] |
| 296 | Lpg2164 | N/A | Q5ZTJ7 | [26] |
| 297 | Lpg2222 | N/A | Q5ZTE0 | [26] |
| 298 | Lpg2375 | N/A | Q5ZSZ1 | [26] |
| 299 | Lpg2395 | N/A | Q5ZSX1 | [26] |
| 300 | Lpg2482 | N/A | Q5ZSN5 | [26] |
| 301 | Lpg2607 | pepO | Q5ZSB2 | [26] |
| 302 | Lpg2907 | N/A | Q5ZRH5 | [26] |

**Reference:**

1. Huang L, Boyd D, Amyot WM, Hempstead AD, Luo ZQ, O’Connor TJ, et al. The E Block motif is associated with Legionella pneumophila translocated substrates. Cell Microbiol. 2011;13: 227–245. doi:10.1111/j.1462-5822.2010.01531.x

2. Altman E, Segal G. The Response Regulator CpxR Directly Regulates Expression of Several Legionella pneumophila icm/dot Components as Well as New Translocated Substrates. Journal of Bacteriology. 2008;190: 1985–1996. doi:10.1128/jb.01493-07

3. Zhu W, Banga S, Tan Y, Zheng C, Stephenson R, Gately J, et al. Comprehensive Identification of Protein Substrates of the Dot/Icm Type IV Transporter of Legionella pneumophila. Kwaik YA, editor. PLoS ONE. 2011;6: e17638. doi:10.1371/journal.pone.0017638

4. de Felipe KS, Pampou S, Jovanovic OS, Pericone CD, Ye SF, Kalachikov S, et al. Evidence for Acquisition of Legionella Type IV Secretion Substrates via Interdomain Horizontal Gene Transfer. Journal of Bacteriology. 2005;187: 7716–7726. doi:10.1128/jb.187.22.7716-7726.2005

5. Kubori T, Hyakutake A, Nagai H. Legionella translocates an E3 ubiquitin ligase that has multiple U-boxes with distinct functions. Mol Microbiol. 2008;67: 1307–1319. doi:10.1111/j.1365-2958.2008.06124.x

6. Burstein D, Zusman T, Degtyar E, Viner R, Segal G, Pupko T. Genome-Scale Identification of Legionella pneumophila Effectors Using a Machine Learning Approach. PLoS Pathog. 2009;5: 12. doi:10.1371/journal.ppat.1000508

7. Shohdy N, Efe JA, Emr SD, Shuman HA. Pathogen effector protein screening in yeast identifies Legionella factors that interfere with membrane trafficking. Proceedings of the National Academy of Sciences. 2005;102: 4866–4871. doi:10.1073/pnas.0501315102

8. Luo Z-Q, Isberg RR. Multiple substrates of the Legionella pneumophila Dot/Icm system identified by interbacterial protein transfer. Proc Natl Acad Sci U S A. 2004;101: 841–846. doi:10.1073/pnas.0304916101

9. Heidtman M, Chen EJ, Moy M-Y, Isberg RR. Large-scale identification of Legionella pneumophila Dot/Icm substrates that modulate host cell vesicle trafficking pathways. Cell Microbiol. 2009;11: 230–248. doi:10.1111/j.1462-5822.2008.01249.x

10. Zusman T, Aloni G, Halperin E, Kotzer H, Degtyar E, Feldman M, et al. The response regulator PmrA is a major regulator of the icm/dot type IV secretion system in Legionella pneumophila and Coxiella burnetii. Mol Microbiol. 2007;63: 1508–1523. doi:10.1111/j.1365-2958.2007.05604.x

11. Pan X, Lührmann A, Satoh A, Laskowski-Arce MA, Roy CR. Ankyrin Repeat Proteins Comprise a Diverse Family of Bacterial Type IV Effectors. Science. 2008;320: 1651–1654. doi:10.1126/science.1158160

12. Ninio S, Zuckman-Cholon DM, Cambronne ED, Roy CR. The Legionella IcmS-IcmW protein complex is important for Dot/Icm-mediated protein translocation. Mol Microbiol. 2005;55: 912–926. doi:10.1111/j.1365-2958.2004.04435.x

13. Conover GM, Derré I, Vogel JP, Isberg RR. The Legionella pneumophila LidA protein: a translocated substrate of the Dot/Icm system associated with maintenance of bacterial integrity. Mol Microbiol. 2003;48: 305–321. doi:10.1046/j.1365-2958.2003.03400.x

14. Xu L, Shen X, Bryan A, Banga S, Swanson MS, Luo Z-Q. Inhibition of host vacuolar H+-ATPase activity by a Legionella pneumophila effector. PLoS Pathog. 2010;6: e1000822. doi:10.1371/journal.ppat.1000822

15. VanRheenen SM, Luo Z-Q, O’Connor T, Isberg RR. Members of a Legionella pneumophila Family of Proteins with ExoU (Phospholipase A) Active Sites Are Translocated to Target Cells. Infection and Immunity. 2006;74: 3597–3606. doi:10.1128/iai.02060-05

16. Ninio S, Celli J, Roy CR. A Legionella pneumophila Effector Protein Encoded in a Region of Genomic Plasticity Binds to Dot/Icm-Modified Vacuoles. PLOS Pathogens. 2009;5: e1000278. doi:10.1371/journal.ppat.1000278

17. Campodonico EM, Chesnel L, Roy CR. A yeast genetic system for the identification and characterization of substrate proteins transferred into host cells by the Legionella pneumophila Dot/Icm system. Molecular Microbiology. 2005;56: 918–933. doi:10.1111/j.1365-2958.2005.04595.x

18. Nagai H, Kagan JC, Zhu X, Kahn RA, Roy CR. A Bacterial Guanine Nucleotide Exchange Factor Activates ARF on *Legionella* Phagosomes. Science. 2002;295: 679–682. doi:10.1126/science.1067025

19. Zusman T, Degtyar E, Segal G. Identification of a Hypervariable Region Containing New Legionella pneumophila Icm/Dot Translocated Substrates by Using the Conserved icmQ Regulatory Signature. Infection and Immunity. 2008;76: 4581–4591. doi:10.1128/iai.00337-08

20. Liu Y, Luo Z-Q. The Legionella pneumophila Effector SidJ Is Required for Efficient Recruitment of Endoplasmic Reticulum Proteins to the Bacterial Phagosome. Infection and Immunity. 2007;75: 592–603. doi:10.1128/iai.01278-06

21. Machner MP, Isberg RR. Targeting of Host Rab GTPase Function by the Intravacuolar Pathogen Legionella pneumophila. Developmental Cell. 2006;11: 47–56. doi:10.1016/j.devcel.2006.05.013

22. Chen J, de Felipe KS, Clarke M, Lu H, Anderson OR, Segal G, et al. Legionella Effectors That Promote Nonlytic Release from Protozoa. Science. 2004;303: 1358–1361. doi:10.1126/science.1094226

23. Shen XH, Banga S, Liu YC, Xu L, Gao P, Shamovsky I, et al. Targeting eEF1A by a Legionella pneumophila effector leads to inhibition of protein synthesis and induction of host stress response. Cell Microbiol. 2009;11: 911–926. doi:10.1111/j.1462-5822.2009.01301.x

24. Ivanov SS, Charron G, Hang HC, Roy CR. Lipidation by the Host Prenyltransferase Machinery Facilitates Membrane Localization of Legionella pneumophila Effector Proteins. The Journal of Biological Chemistry. 2010;285: 34686. doi:10.1074/jbc.M110.170746

25. Lifshitz Z, Burstein D, Peeri M, Zusman T, Schwartz K, Shuman HA, et al. Computational modeling and experimental validation of the Legionella and Coxiella virulence-related type-IVB secretion signal. Proc Natl Acad Sci U S A. 2013;110: E707–E715. doi:10.1073/pnas.1215278110

26. Burstein D, Amaro F, Zusman T, Lifshitz Z, Cohen O, Gilbert JA, et al. Genomic analysis of 38 Legionella species identifies large and diverse effector repertoires. Nature Genet. 2016;48: 167–175. doi:10.1038/ng.3481
