# Supplementary material for: Legionella effector LpPIP recruits protein phosphatase 1 to the mitochondria to induce dephosphorylation of outer membrane proteins
Source: PLoS Biol. 2025 Jul 23;23(7):e3003261. doi: 10.1371/journal.pbio.3003261 (PMC12313075; doi:10.1371/journal.pbio.3003261)
Supplement: S2 Text — (DOCX) [file pbio.3003261.s013.docx]

>tr|Q5ZZG9|Q5ZZG9_LEGPH Peptidase M12B domain-containing protein OS=Legionella pneumophila subsp. pneumophila (strain Philadelphia 1 / ATCC 33152 / DSM 7513) OX=272624 GN=lpg0041 PE=4 SV=1

MILLNLMGFTMIRTFLTAFLMLLCSLALAQEKVVTQFFKEVNSSIAISEGKANIQASRYRVVDIDVNQLYAELENAPHRDGISTGIPLQLELPQPDGTVKRYQVMENSTLAPELSTKFPEIKTYDAYGIDNPGELVKFDLTPQGFHAMILSPGRDTVFIDPLIKGNTQYYMIYYKKDFITSKKMKCGVKNQNQPLINAASKRDFTDFNPCVLKKYRLALAATAQYTQFHGGTVPQALAAEATTVNRVNGIYEIDMAITMQIIANNNLIIYTDPNNQPYTSGDPDKMIGENQANIDKEIGAANYDIGHVVDAAGSGLAQTPSVCINGEKAMGVTGQSNPVGDPFDVGYVAHEIGHQFGANHVQNNNCQRNNPTAVEPGSGSTIMSYAGICDPNVQDDSDPYFNGISLQEMGNFVSDATHTCPVKTSIPSAPVIQGTNGGVKIPAQTPFALTATATKSGGNEALTFAWEQQNNEASQQPPVSTSRVGPNFRSFSPQVSGTRYFPNLNALANNGPFTWEVLPSVSRTLKFRVTVRRNTPGGSCNAYTDTTLSVESKAGPFVVTNPTESGITWTGISP**RTVTW**DVANTNLPPINARFVNILLSIDGGQTFPFTLLSNVDNTGSEVICVPNLNSSTARIMVQASNGTFFNVSKNNLTIIPVPPRPPELTRADRNPMDTSKAFILYANCIPTGNDVYTVNGLPPGATVRFDANNRRFIIENINTPKRVRNVTITATDENGVSRTSNAITIPSIL

>tr|Q5ZYG7|Q5ZYG7_LEGPH Coiled-coil protein OS=Legionella pneumophila subsp. pneumophila (strain Philadelphia 1 / ATCC 33152 / DSM 7513) OX=272624 GN=lpg0405 PE=4 SV=1

MLYNLT**RQVKF**NNPSCLCNFQKMGYIDELLEELSARLPELEWKINGLSSSISIHNLPKGIFSSVIEFNGPACIKEIQDDIHALQKHKDESIACFLAERIQKKINVLVVLCQMDKKNNKPESKISFDLTTLSTRQQWIKTMEEKICALEEQYQAMRKTLEQMKSCSNPATILHLQTELGNVEKRLTLAKETLNRAIS

>tr|Q5ZWC7|Q5ZWC7_LEGPH Type IV secretion protein Dot OS=Legionella pneumophila subsp. pneumophila (strain Philadelphia 1 / ATCC 33152 / DSM 7513) OX=272624 GN=lpg1158 PE=4 SV=1

MIIYLSIWLIIGMKELIAFIHDLYKDRTE**KSVKF**ESKGRVIVSPLMSKDDAQELFKSLKEILPEEARLKVRKSKQEPDQFRVVLFNPEKFFSFYAEHAAELLNRFKPLPTSSSGEEYCKWKYNPLSHLIEYTVPFSILDSDTKAKVTDTHEEAIQSKVNQYNEKLRSIYPNLKIIGTNSVEDSSPDKFFLGSFNYNDYKIIKNLNNPASLRQLAIGFFKEHPGHYTEEVAKKIPGDMKDEFTDIEPSSTKPSSTGS

>tr|Q5ZV52|Q5ZV52_LEGPH Coiled-coil-containing protein OS=Legionella pneumophila subsp. pneumophila (strain Philadelphia 1 / ATCC 33152 / DSM 7513) OX=272624 GN=legC6 PE=4 SV=1

MSHSKREQNPIARNLSDFRQVATLLNNEGLVVTEHNYKKVQALLNRYKELATFIDQNQSKLNGKQIGELNQIRLIISPEERREIRKSVTRIENGIKNREERIKEEYTRIEKQQAKAREVVQVLWPQLQKAAKKFINSGIANMEELLKEYKDLRNELYNHALNLPTQERKQKNQEMAELSRLVNIHESKLYEEINRARNVIEAAVTQAQKNVDSMSLLQMVRLMLPKLKDADILKIAQKAGVLSLQDTTTSEHGANLDKLVVREQPLEKQSALISIMFKELALNGAQAGPLAKYLQAVQENAKDLISGKKTHFTLLYQTNEGEIGGFYFNIHELLQNQHQAISKLVHEMYKRGVDQNSRVVMAFASPEQTASLMLANGENIKALTEAGNLQELTEQLLENAGDSKALARIVGHDVVIGQKTSEIIRDLNRSKEQNGIEPAEVMKLFQIIAVIFETRNILINWITAPVHLKEQTEEALLGAGKTPAIVEGKGTQP**KRVRF**LPDLVEKPKATSPDTEQPVTKRKDDELLDKKIQVALEKVDQALEELKGKIGDINQHKYDKAYEAAKILLRQLQETRDQYAIDLANPQINFKEAGLKFKEASNLVIQQAKPILEKDLGWGEYLKNLFKVIVNAIVFCVTFGASQGFFATTRAKSAEAVEKAESELELNQLGSLPK

>tr|Q5ZV17|Q5ZV17_LEGPH Uncharacterized protein OS=Legionella pneumophila subsp. pneumophila (strain Philadelphia 1 / ATCC 33152 / DSM 7513) OX=272624 GN=lpg1625 PE=4 SV=1

MQNEKEVKELIQSDKK**KRVTW**NDNQNEGQIDEKFLKIGTASTPYKNKLDESIAQLMEEGYSEIEATAITGKSVTPAGMVIYNNPVFYKSKESIDIEKANRYNFFSIKNIGLGIVALAAAATVGLVVSKNS

>tr|Q5ZUS5|Q5ZUS5_LEGPH Type IV secretion protein Dot OS=Legionella pneumophila subsp. pneumophila (strain Philadelphia 1 / ATCC 33152 / DSM 7513) OX=272624 GN=lpg1717 PE=4 SV=1

MPVRYSGFDYDGCVGVTNLINIVAQNPKLAQRIRETSNYYTKEVALIASNRQSDRDDKRNARSNGNGSCFIQIQKLCTDLGMEFDPFLLADAYNLVPAGTSLHEATNPANGAKIQNGWLHDKSKASLVYAHMQRASSLEAREQIDYFFYDDREDILEGLYQFFSANPELIPSNVTMHLVKYACSRENVHTMDNYKIPVQGKGFTNPDYVKTIKAFGDKAIENARDVYDIDGKEINAAGVRQAKYNCNFEFEANKLQPMKFVNNFNSPAELIKAHEQAIEKIKLGAFELYKAKINVITTLSNEQKVFLIRAKAQQWNLLCDEGQWNNPIFTLQAIMAEKSDRFFLPQYAFTAKMLIKNYQEARENSKDGSRFWEQINAFIKSMNALKDPMRNAQSIFEVFKKNSYSVKAVDGFVKKVVEVMNALMDKYPQRSTTEMVACMRILESNLQ**KMVNF**FSLVDRLITIYRGCTTAGLNFNKILTVVREALEEMKMITDSGDNYSTTLIDLDKVITAANNRLESLTYPKEYSTKNFHSQESYKVVGAYLCMQSQLIQKLDTKASTALAI

>tr|Q5ZU83|Q5ZU83_LEGPH Protein kinase domain containing protein OS=Legionella pneumophila subsp. pneumophila (strain Philadelphia 1 / ATCC 33152 / DSM 7513) OX=272624 GN=lpg1924 PE=1 SV=1

MPLNLPPKSSKNTMPLVIAYNNAPEDDKIQKLFYLQKINYLLNKTQLNDDLFDWINDAEEGGWLNELAKFSINPNASFFLKGMQFAKAITEEIKNKPEINSSEVNIYHLMQERDQLLKEVEFEKCATRYAEINFLLNELALNDKKTKEIVERQTEILRLVAPKIKAIKGESIDNLPVIPSYKTKELGNHVNNFNFKFTMSGWEAPFVFRVEDRHELGKEQELHSYGVSKYFIEDYSVFMMRFKAEDGSTVYKPVILSQFANQNNLEEIAKQLKDGSPKNIAPRIGYYFVQLTDFCLKLIETHNYHPDIKLNNFLVHNNRVLVSDRKTFTTNDNPLASEILTSPLFAPDEFLKCLLFNKEGDPVGYNRNALWKRMNMPQFMAYQLGMALKQFLILTQLDELPDDFRNPDHSAVSHFKTPSRQIINLSLLVQELTRLDPDKRMTIKQFQTLLNFKNLPPDAFYQKVEEVFPSSQLGIAEDIEALNKVLNSDLKGEALLKQANPVFTKLSKYDPKETRLTRLAEKLAIRCFNNVSKPYFQKLSGLIESALEKDVNEIRQLLNPQSNSERLLQKASPIFMKLFERQPEVPQLTDLAKQLAAQCFDESSKNYFTRQLPLLIEEELLNQDWEQAPWYRKALHWLTFGYFRVDNVTEISSLKIPEHTKGKEFQIYFWQLLFLPSEEIKNMFTSKDSQISEFLLGYIEKEREKRETETPSVQIPVDLKGEEFQIHFPQLEFLPSKDFESIGEKEGEHLECFIFANLQEILSHNNSDSSEKNSTSEPSDSESEIHLNAGNVDTIIIANEPNPTDAKPQKKDNDGAKQNNPAKASSPEGIVSTPEVEEHPKKKITDDAPKPKTK**RSVHF**FDEAKKSKPKEKENASPAVDETPPAKRKNVCRIDSVRSTLFRGDGSHRQKIKEQRPRLSEIAWEPPKPNLQ

>tr|Q5ZSK9|Q5ZSK9_LEGPH SidJ OS=Legionella pneumophila subsp. pneumophila (strain Philadelphia 1 / ATCC 33152 / DSM 7513) OX=272624 GN=lpg2508 PE=4 SV=1

MFSYLDKLLDGIFGYQKSDTSQLSTSTPPTLTTVPLKQEYFVKIGESETQDLGLLPVVSKRHNQSVPLEEIPFEDTRLELIELYIALLKQCVLDEKLKSIPAQYLISHYLFIKTLAANEGNKGRKDLYLNLSQKVADYLEKNESKIWSMAVECAKTSEYPIVDWIKKHHLHFNFIRAFILDYNKKSLTHNQRAFMQQFRDSAAFFFPDQVYLAWLTQSYEPGSILNPMYRESRSTHYYHANITDNLLLRTRP**KQVNF**GPQHFFQKGKGPVKNTYRFNINDGKLMRIQGRTLLFSTNKGNEVIAVKVQKKGEPQSALSNEFQMADYLLKHQRRLNLQSQLPTPLSQYSINRTEILEKCSKSPDFEKFKNLISDAKSLEIYVYKATPSYFTYLHDKQQSFSQLTSSVQKNVHDLFVLLREGIVFPYLADMFHTHIDESKRSDKGRYQTLVELLSALQSQMGRLDKWQKAVEFVNLRASGIADLGDNLPLTSFLTVSDWTKHYHAELLTGVYHPSFLFLDKSSGSVRSLFNSRRKIFGNYLYLNIIAEYLLVIQLVIGCYGDKVTRKMNSKSKAKVWEHLAELMFSSCAEAVNLITGMPKSRALAFLKQRANVRKHTQQTSFWMTPDYSNLDRLSVQSQQSTLYPGESDYEINSDLISGVGLSLDGINQDLGDYNQASPLRELEKLLYATVTLIEGTQQLDKQFFQQLDETEKMILSAAGVDKCYQAVAKLLDLARPGCQMQRRLAFTYYEMIKRIYPCSNPADVRFELVAKEEAIIKIQRFWREHKKENQSLEKGFDFDRNTSSSQSPL

>tr|Q5ZSH9|Q5ZSH9_LEGPH Uncharacterized protein OS=Legionella pneumophila subsp. pneumophila (strain Philadelphia 1 / ATCC 33152 / DSM 7513) OX=272624 GN=lpg2538 PE=4 SV=1

MIPVPVMRKTHELVMIQAAAGFFMKTKYEMSQDKTEFLAKEQDSSYPGYQVSVLDLEKIVKHYQEKYGIRLIINGTTPKYQSLIKE**RQVNF**EQQKQQFLELKYAKFLQIFFQPPNLNGANSPFSINKHMGAFIGFYEEIYNKVLPFLDAKGKIISGLSLEELRQLNEACQELSCKGILDAKINEFIERNFDYMGLTARESASEIKDICDELQEGEVLGYFFTGQRTSGRCHFDLYICLPGKAIRPIFYNTALIRYHDLGGMFHLNFPFVEGNFFTPDLLKLYSAMDLQQLIPQADRTSCGTLTMMYAKELLKDDARGLKEFTLSFTYYNDKGEKEYFFLPSPQVLRYSQISLYNEALKAILSHENDGQAGLVRKGAKKYMFHTVEKILIQSFKIALEKEDADVLEENQKIWDILPSFQEKWQEAYKEMVAKRDVMHQGVNKYLLYSTHRMSHIASDQSINNEADADRLILR

>tr|Q5ZRP9|Q5ZRP9_LEGPH VipD OS=Legionella pneumophila subsp. pneumophila (strain Philadelphia 1 / ATCC 33152 / DSM 7513) OX=272624 GN=vipD PE=1 SV=1

MKLAEIMTKSRKLKRNLLEISKTEAGQYSVSAPEHKGLVLSGGGAKGISYLGMIQALQERGKIKNLTHVSGASAGAMTASILAVGMDIKDIKKLIEGLDITKLLDNSGVGFRARGDRFRNILDVIYMMQMKKHLESVQQPIPPEQQMNYGILKQKIALYEDKLSRAGIVINNVDDIINLTKSVKDLEKLDKALNSIPTELKGAKGEQLENPRLTLGDLGRLRELLPEENKHLIKNLSVVVTNQTKHELERYSEDTTPQQSIAQVVQWSGAHPVLFVPGRNAKGEYIADGGILDNMPEIEGLDREEVLCVKAEAGTAFEDRVNKAKQSAMEAISWFKARMDSLVEATIGGKWLHATSSVLNREKVYYNIDNMIYINTGEVTTTNTSPTPEQRARAVKNGYDQTMQLLDSHKQTFDHPLMAILYIGHDKLKDALIDEKSEKEIFEASAHAQAILHLQEQIVKEMNDGDYSSVQNYLDQIEDILTVDAKMDDIQKEKAFALCI**KQVNF**LSEGKLETYLNKVEAEAKAAAEPSWATKILNLLWAPIEWVVSLFKGPAQDFKVEVQPEPVKVSTSENQETVSNQKDINPAVEYRKIIAEVRREHTDPSPSLQEKERVGLSTTFGGH

>tr|Q5ZZ03|Q5ZZ03_LEGPH Serine/threonine-protein kinase OS=Legionella pneumophila subsp. pneumophila (strain Philadelphia 1 / ATCC 33152 / DSM 7513) OX=272624 GN=pkn5 PE=4 SV=1

MCNHSLNEPHLGSSYLACESMFGSYYFALYFALNFFLMIVFYNAKVIFIQVCKYVRFLMKLLRFHELKSLPSMDEKALELLIKVLGNKGIRKLIKSADGKPISREIMIHEFGIDCQILFITTEASLKPIIVPTENKISGGGKSYCEQFKVYALDDGKTYFLKSVKINAESLTEFTNETDTLSKLGRLVGTFFNEQTQVHYILTTFIKGIDLSRYKNALPLNINLKHFWEVLGIMISVCHQVKQFHELGLIHRDLKPGNIMLDADMQCHLVDFGSSSSDKEPKPVSWGTASYLAPELNAQEDFIAFSQASDLFALAYSLDELFNPF**RQVKF**AKVDIGIKNKHLVLLHAEIEACITGLMSNETSVRTLYFSRILQLQRVPESFKSRPEAFTYMIMLLTQWKSCYEAPEMNKELDEIIAEIKVAYENHEQDVVKIITLLEQLSKADGLLNSHKALLSVLIKSLANVQQQELGQDDILPRRFESDVVSRLIKTPTAKMMAAIKQVSDAIVKILEQYEHSPAESFEHAVLQDFLEQMVQSDILFGAPKEKIEISDIIKILKANRPEDFVQIQHISFKFAQVALRDLALHDLPKHEPKGAFLEILKKYQAKYAPEETSLFELYHHMAGVRDHNPSKVKKGEGYRFRAFICDELFYGDEIFTTGDNRGREGKPNQRLQTNQVGLMKFEHSAHTKGLLTLNGQSWYADCKTQLPNYDSIHYLSALKTDCPYITGPSGMTSLFMNMMFLLLNPKDQDVILSYSLGVMTYVVGAGYHSIKEILIPMVKCVGIVPDYPQHKGTECLTAPPLYNHYFKAIEEFDKEFAEVHEKIWQEYLAYFRMTYMPVCMRSHCLPHQVPSMEDISSEVKEMLEIVSKSVKNCLEEHKVKRTGDMGLFLSTPCGQTKALEVLSHVCQQQVGLTAIFRQIQAYFKGTFETEEGIIINREMQLKFIAHFFDVLKESPTLLSQFNLTLRIENTIGVHQCELGSSKQKELFLEIKKTSIAANEEQMVANHEPRKENRNVIILPY
