# Supplementary material for: Legionella effector LpPIP recruits protein phosphatase 1 to the mitochondria to induce dephosphorylation of outer membrane proteins
Source: PLoS Biol. 2025 Jul 23;23(7):e3003261. doi: 10.1371/journal.pbio.3003261 (PMC12313075; doi:10.1371/journal.pbio.3003261)
Supplement: S1 Raw Images — (PDF) [file pbio.3003261.s018.pdf]

Fig 1D:

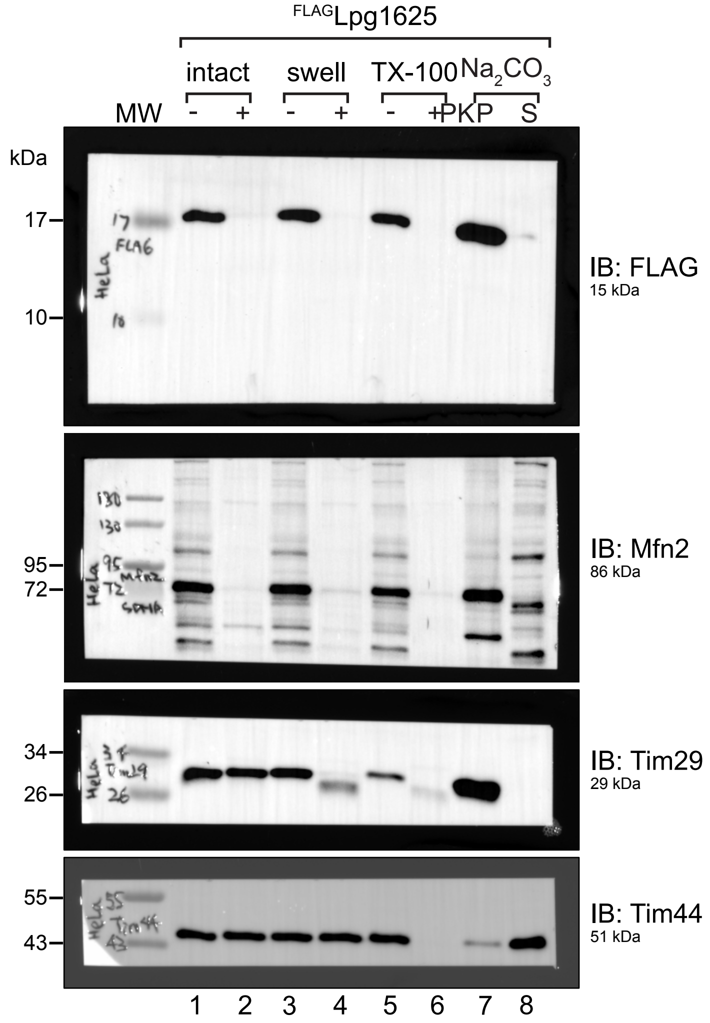

Western blot image acquisition: Western blot images were acquired using a Bio-Rad ChemiDoc™ Imaging System. Images are presented as composites combining chemiluminescent signals (ECL substrate) and colorimetric signals used to visualise the protein ladder. Both channels were captured from the same blot and assembled using Image Lab software to overlay the molecular weight markers onto the chemiluminescent image.

Fig 2C:

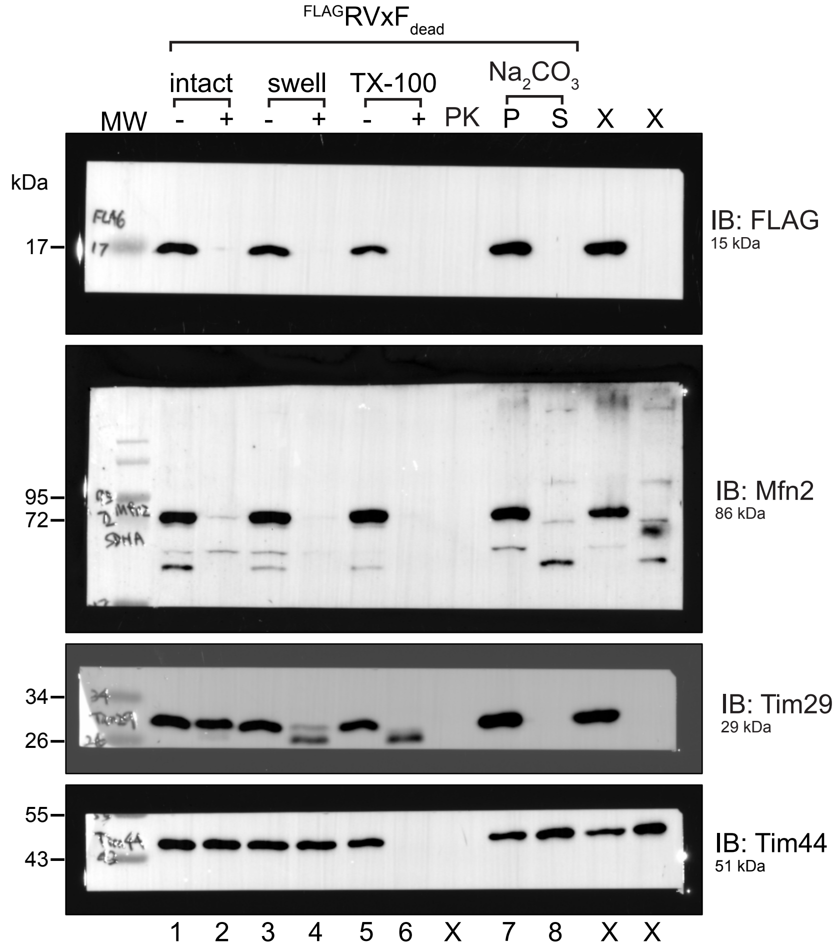

Fig 4C:

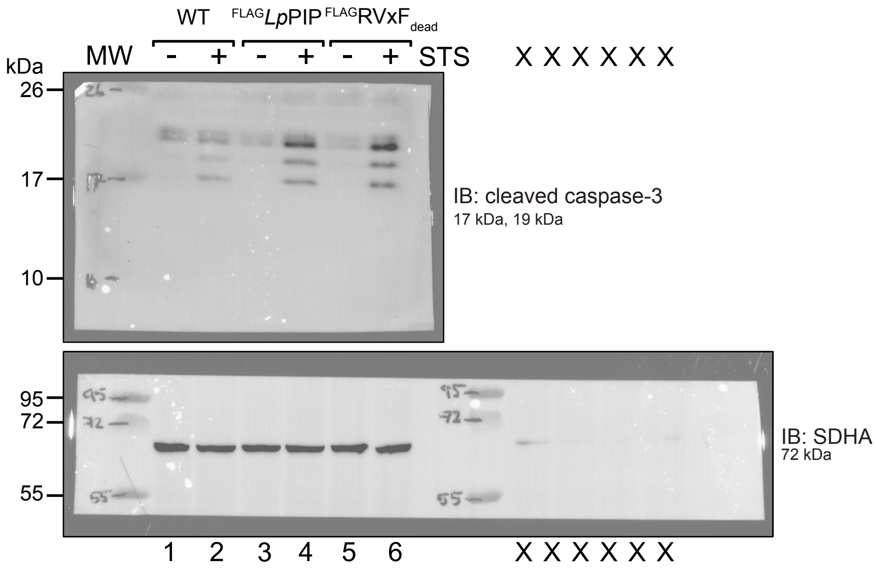

Western blot image acquisition: Western blot images were acquired using a Bio-Rad ChemiDoc™ Imaging System. Images are presented as composites combining chemiluminescent signals (ECL substrate) and colorimetric signals used to visualise the protein ladder. Both channels were captured from the same blot and assembled using Image Lab software to overlay the molecular weight markers onto the chemiluminescent image.

Fig 4H:

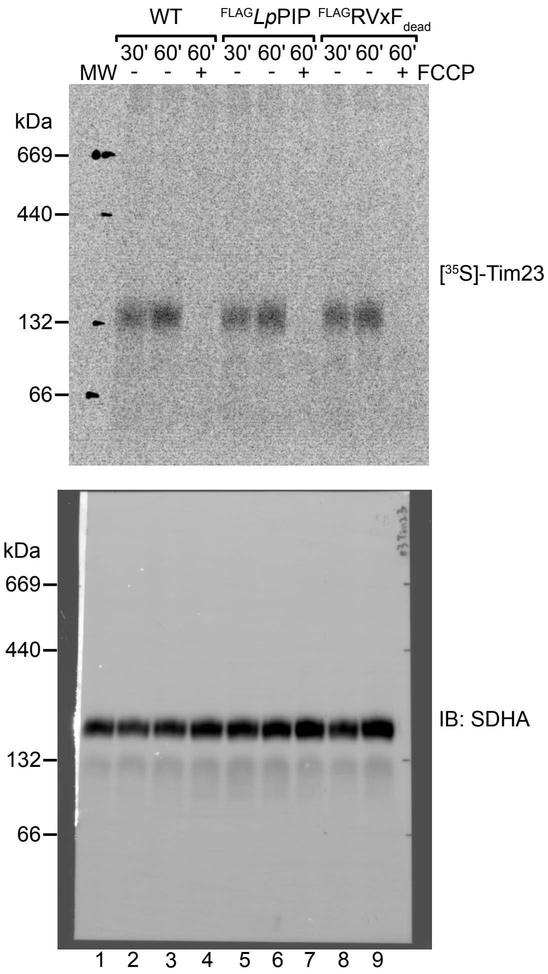

Fig 4I:

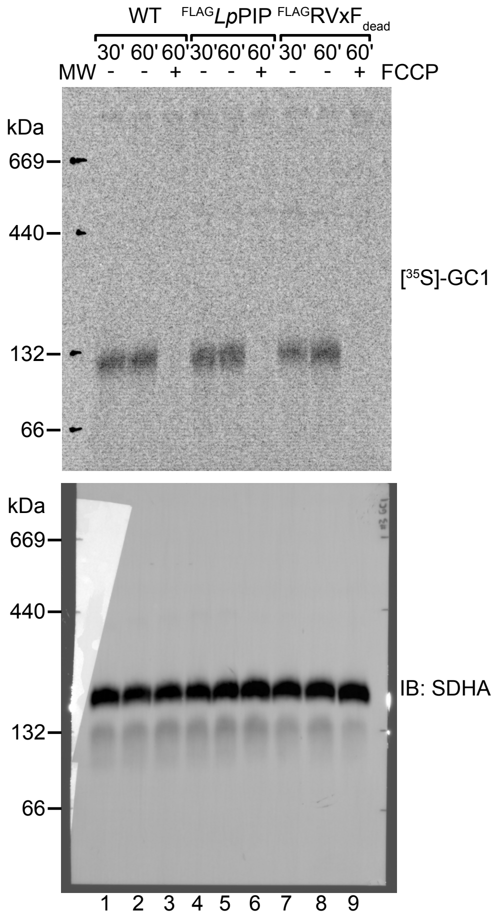

Detection of radiolabelled proteins: After BN-PAGE and transfer to PVDF membrane, radiolabelled proteins were visualised by exposing the membrane to a storage phosphor screen (GE Healthcare) within a light-tight cassette for 11 days. The screen was then scanned using an Amersham Typhoon phosphorimager (GE Healthcare) to detect the radioactive signal.

S2F Fig:

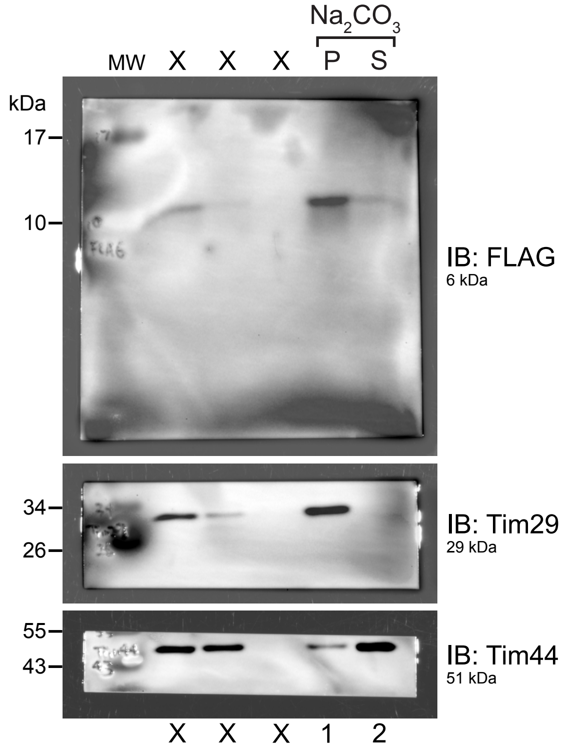

S3A Fig:

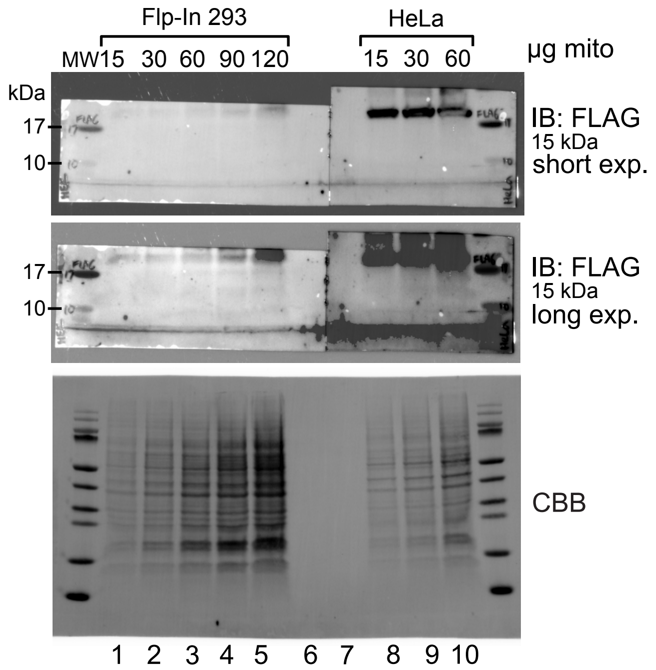

S3B Fig:

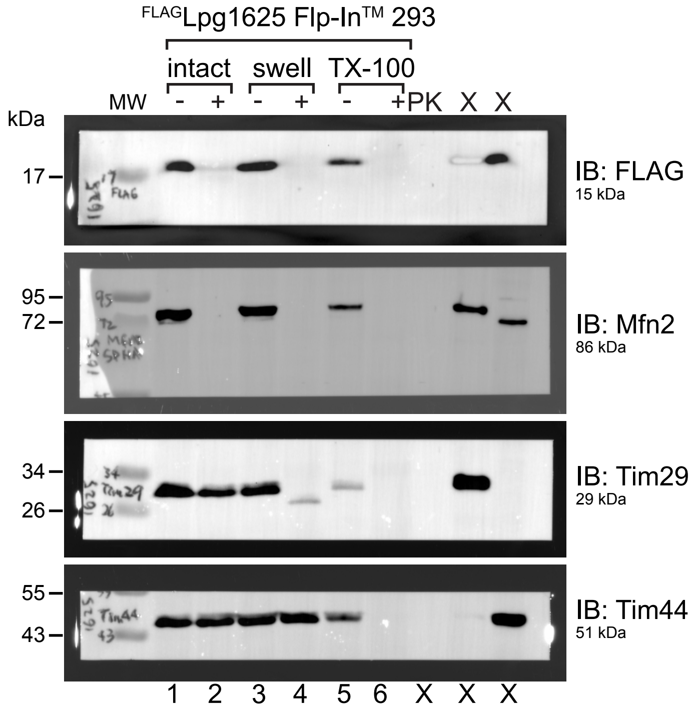

S3D Fig:

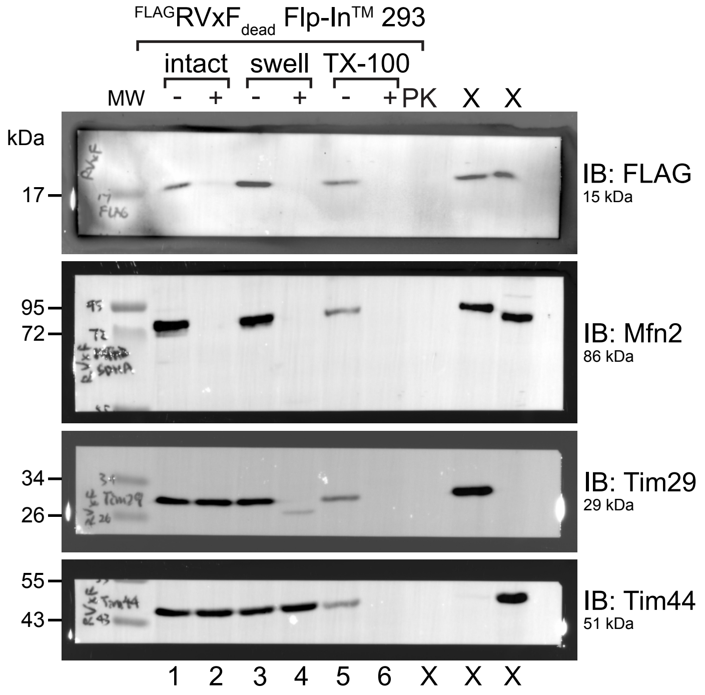

Western blot image acquisition: Western blot images were acquired using a Bio-Rad ChemiDoc™ Imaging System. Images are presented as composites combining chemiluminescent signals (ECL substrate) and colorimetric signals used to visualise the protein ladder. Both channels were captured from the same blot and assembled using Image Lab software to overlay the molecular weight markers onto the chemiluminescent image.
