# Supplementary material for: Legionella effector LpPIP recruits protein phosphatase 1 to the mitochondria to induce dephosphorylation of outer membrane proteins
Source: PLoS Biol. 2025 Jul 23;23(7):e3003261. doi: 10.1371/journal.pbio.3003261 (PMC12313075; doi:10.1371/journal.pbio.3003261)
Supplement: Attachment — Submitted filename: Yek et al_Response Letter.docx [file pbio.3003261.s021.docx]

**A/Prof. Diana Stojanovski**

Department of Biochemistry and Pharmacology

The University of Melbourne

The Bio21 Institute, Melbourne, 3052, Australia

E: d.stojanovski@unimelb.edu.au

**Comments highlighted by Editor**
[1] Reviewer 1 thinks it’s unclear what is the downstream consequence of the dephosphorylation on mitochondrial biology and cell survival, and that it would help to show that not all organelles show a similar overlap with Lpg1625-FLAG staining as a control. This reviewer also asks for several clarifications and a discussion of the limitations of the study.

**Point 1:** **New Figure 4** addresses this comment with extensive additional experiments, looking at mitochondrial pathways in wild-type cells versus cells expressing ^FLAG^*Lp*PIP and ^FLAG^RVxF_dead._ Pathways analysed included:

- Mitochondrial respiration measurements assessing oxygen consumption rate (Fig 4A).
- Cell death induction by looking for cleaved caspase 3 (Fig 4C).
- Mitochondrial morphology, specifically looking at mitochondrial length and network complexity (Figs 4D and 4E) and recruitment of the dynamin related protein, Drp1, to mitochondria (Fig 4F).
- Import of Tom70 substrates (one of our dephosphorylated substrates) (Figs 4H and 4I)
- Basal mitophagy quantified with mt-Keima and live-cell microscopy (Fig 4J)

This analysis did not uncover any obvious difference due to the expression of ^FLAG^*Lp*PIP. This lack of a functional consequence in our cell models could be due to numerous reasons and this is explored in the discussion. Potential possibilities for the lack of phenotypes being observed are explored in the discussion:

**Line 332:** *Indeed, since we did not observe any functional consequences at the mitochondria, it raises the possibility that the bacterium may deliberately sequester PP1 to this organelle to divert the phosphatase away from the nucleus or cytosol. Although we did not have the opportunity to explore this possibility, it remains a compelling hypothesis that warrants consideration.*

**Line 352:** *The downstream consequences of the targeted dephosphorylation of mitochondrial outer membrane proteins identified in our study remain elusive. It is plausible that the presence of L. pneumophila infection may be necessary to reveal relevant phenotypes, if LpPIP-PP1 targets other effectors localised to the mitochondrial outer membrane. The functional impact of LpPIP-PP1 activity may be shaped by interplay with additional effectors. For instance, the T4SS effector LnaB catalyses AMPylation of phosphorylated residues, adding an AMP moiety to the phosphate chain to generate a novel post-translational modification termed ADPylation [85]. This additional layer of regulation could influence the susceptibility of modified substrates to PP1-mediated dephosphorylation. As mentioned above, LpPIP-mediated PP1 re-localisation may exert broader cellular effects by altering phosphorylation of cytosolic or nuclear proteins.*

Despite these results, the striking overlap between our phosphoproteomic data and previously reported phosphosites on mitochondrial proteins during Legionella infection [1] strongly supports the biological relevance of PP1 recruitment by *Lp*PIP. Our work has uncovered the specific phosphatase responsible for remodelling proteins at the mitochondrial outer membrane, providing a critical mechanistic link. Future research will aim to distinguish which of the proposed scenarios outlined in our discussion accurately reflects how Legionella manipulates host mitochondrial signalling.

**Point 2:** Assessment to show that *Lp*PIP overlap with other organelles. For this, we transfected HeLa cells with ^FLAG^*Lp*PIP and stained these cells with FLAG antibodies, and antibodies to other organelles that we know are home to TA proteins. This included Mitochondria, ER, Peroxisomes and Golgi (**new Figs S2A-D**). Co-localisation between the FLAG signal and mitochondria, ER, peroxisomes and Golgi has been quantified. This shows a specific localisation on ^FLAG^*Lp*PIP to mitochondria only.

**Point 3:** Limitations of the study are now included in the final paragraph of the discussion:

**Line 373**: *A key limitation of our study is that these findings were derived from ectopic expression systems in the absence of infection. Given that* L. pneumophila *translocates hundreds of effectors and triggers extensive host cell reprogramming during infection, the function, localisation, or substrate specificity of LpPIP may differ in that context. Future work will be critical to validate these observations under infection conditions and fully elucidate the physiological relevance of LpPIP-mediated phosphoregulation.*

[2] Reviewer 2 mentions that you could also test whether Lpg1625 also targets peroxisomes and perform a co-staining with an ER marker to confirm that the Lpg625 charge variants are targeted to the ER. In addition, this reviewer suggests checking if the C-tail is sufficient for mitochondrial targeting and membrane integration by subcellular fractionation.

**Point 1:** See Point 2 above (Reviewer 1) (**Figs S2A, S2B, and S2D**) we confirm no localisation of *Lp*PIP with peroxisomes.

**Point 2:** HeLa cells transfected with the *Lp*PIP charge variants and were stained with PDI as the ER marker, with the images now included in **Fig S2E**. The quantification of the co-localisation of these charge variants with both mitochondria and ER showed that they indeed colocalise with the ER marker, but also partially localise to mitochondria (**Fig S4A**). This indicates that the loss of positive charge flanking the TMD disrupts the correct targeting of these charge variants to mitochondria, resulting in partial mis-localisation to the ER.

**Point 3:** Mitochondria isolated from HeLa cells transfected with ^FLAG^*Lp*PIP^85-130^ (the TA only construct) were subjected to proteinase K treatment and carbonate extraction. This showed that the TA only construct is localised and embedded in the mitochondrial outer membrane (**Fig S2F**).

[3] Reviewer 3 makes several suggestions for improvement, including checking if there are quantifiable differences in morphology between wild type, LpPIP and RVxF dead cells.

We now include data demonstrating that mitochondrial morphology (length and network) is not different between HeLa cells expressing ^FLAG^*Lp*PIP or ^FLAG^RVxF_dead_ (Figs 4D and 4E).

Specific comments from Reviewers:

**Reviewer 1**

[1.1] While this paper nicely showcases the effect of Lpg1625 on the localization of PP1 and the subsequent dephosphorylation of mitochondrial proteins, it is unclear what the downstream consequence of this dephosphorylation is on mitochondrial biology and cellular survival. The authors discuss PP1 action on Bcl-2 and the role of FUNDC1 in cell death, but does the expression of Lpg1625 alter cell survival? Or mitochondrial parameters such as respiration or morphology?
As outlined above in response to the Editors comments we include a new Figure 4, which shows new experiments to investigate the downstream consequences of *Lp*PIP directed phospho-remodelling at the mitochondrial outer membrane. These included:

[1] Seahorse analysis.

[2] Caspase 3 cleavage: Zhu et al. [2] showed that GFP‑lpg1625 transfected HEK293T cells induced cleavage of caspase‑3. However, the extent of caspase-3 cleavage in our Flp-In™ T-REx^TM^ 293 stable cell lines treated with staurosporine is comparable, indicating that Lpg1625 ability to recruit PP1 does not alter cell survival.

[3] Mitochondrial morphology (length and network) as requested by Reviewer 1.

[4] Basal mitophagy was quantified with mt-Keima and live-cell microscopy.

[5] Import of mitochondrial carrier proteins which are Tomm70 substrates.

As described above in the response to the Editor we have discussed potential reasons for why we have not been able to pinpoint a specific functional consequence at mitochondria and hope the reviewer can appreciate the novelty of the finding in its current format, and that hopefully future work will allow us to distinguish which of the proposed scenarios outlined in our discussion accurately reflects why Legionella recruits PP1 to the mitochondria.

[1.2] While the evidence presented supports a mitochondrial localization, it would be good to show that not all organelles show a similar overlap with Lpg1625-FLAG staining. This should also be quantified.

HeLa cells transfected with ^FLAG^*Lp*PIP were stained with FLAG antibodies, and antibodies to other organelles, including Mitochondria, ER, Peroxisomes and Golgi (**new Figs S2A-D**). Co-localisation between the FLAG signal and mitochondria, ER, peroxisomes and Golgi has been quantified. We have added the following in text to describe the Figure.

**Line 122:** *First, we confirmed that ^FLAG^Lpg1625 localises exclusively to mitochondria and not to other organelles typically targeted by TA proteins (S2A‑D Figs)*

[1.3] The authors argue that, when recruited to mitochondria, PP1 is active and dephosphorylates specific targets. Does this reduce the PP1 activity at other subcellular sites? What fraction of PP1 is recruited to mitochondria?

Whole cell phosphoproteomics presented in **Fig 5E** showed an increase phosphorylation of nuclear proteins in HeLa cells transfected with ^FLAG^Lpg1625 — shown in the GO enrichment analysis in **Figure S4G**. This is similar in HeLa cells transfected with ^FLAG^*Lp*PIP-CYB5A^TA^ (**Fig S4H**). This suggests a reduction of PP1 activity at nucleus. This was elaborated on in the manuscript:

**Line 332:** *Indeed, since we did not observe any functional consequences at the mitochondria, it raises the possibility that the bacterium may deliberately sequester PP1 to this organelle to divert the phosphatase away from the nucleus or cytosol. Although we did not have the opportunity to explore this possibility, it remains a compelling hypothesis that warrants consideration.*

In terms of the fraction of PP1 recruited to mitochondria using Fiji quantification of PPP1CB proportion at mitochondria in HeLa cells expressing ^FLAG^*Lp*PIP, we confirm around 17% of PPP1CB is recruited to mitochondria. We did try and explore this using biochemical means, but found hard to isolate mitochondria pure enough so that this could be quantified in a reasobale manner.

[1.4] The authors claim that binding to Lpg1625 does not prevent PP1 activity because it occurs outside of the catalytic site. Can they rule out allosteric inhibition?

We appreciate this question and have now included this limitation in the manuscript text.

**Line 200:***The model suggests that the PPP1CB active site, which is located at the intersection of the potential substrate binding groove, remains accessible in the complex (Fig 2H) [3]. This suggests that PP1 retains its catalytic activity when bound to LpPIP, although potential allosteric effects cannot be excluded.*

[1.5] For Fig. 1E, the authors argue that localization of the K128A and K97A/R100A/K128A mutants "showed localization reminiscent of the ER" yet they did not co-transfect any ER marker which would prove this association; could this be added?

This has now been confirmed through co-staining with PDI and quantification. HeLa cells transfected with the Lpg1625 charge variants were stained with PDI (**Fig S2E**). The quantification of the colocalisation of these charge variants with both mitochondria and ER showed that they indeed colocalise with the ER marker, but also partially localise to mitochondria (**Fig S4A**). This suggests that the loss of positive charge flanking the TMD disrupts the proper targeting of these charge variants to mitochondria, resulting in partial mislocalisation to the ER.

[1.6] The discussion section would benefit from the authors also discussing the limitations of their experiments / the model that they are suggesting.
Addressed in Point 3 of response to Editor.

Minor concerns / comments:
[1.7] Labelling of the y-axis in Fig S1 does not completely match the figure legend.
This is now fixed.

[1.8] In Fig S1, the authors make use of a FLAGRVxFdead mutant yet creation of this mutant is only explained for Fig. 2C. I would have preferred to see this explanation earlier in the text.

To maintain flow of the manuscript we have added a brief description of this mutant to the figure legend for Fig S1.

*Differentiated THP-1 cells were infected with L. pneumophila JR32 (wild-type), Δlpg1625, Δlpg1625 complemented with ^FLAG^Lpg1625 or* ***^FLAG^RVxF_dead_ (^FLAG^Lpg1625^K17A/V19A^, mutant that loses interaction with PP1)*** *with multiplicity of infection (MOI) of 5 (n = 5 independent experiments). The mean ± SEM for fold change of the colony-forming units (CFU/mL) of the different L. pneumophila strains from THP-1 cells infected over three days against 3 hpi were plotted. Two-way ANOVA was used for statistical analysis.*

[1.9] Following Fig 2, the authors switch from HeLa to HEK293 cells. Could they please explain the reasoning behind this?

We initially performed our experiments in HeLa cells as a robust and well-characterised model system to establish the mitochondrial localisation and functional impact of the effector. However, knowing that transient expression of proteins due to transfection can result in high expression we wanted to validate our findings using the Flp-In^TM^ T-REx^TM^ 293 system, which creates tetracycline-inducible cell lines where protein levels are regulated. Importantly, these two systems mirrored each other, supporting the accuracy of our findings.

[1.10] For Fig. 3D, the authors list n = 4 and n = 3. Is this the number of cells or experimental repeats?

We apologise for the confusion here. This has now been clarified in the figure legends as technical replicates.

[1.11] The authors argue that Fig S2A confirms Fig 1D regarding the localization of Lpg1625 on the outer mitochondrial membrane. In Fig S2A however there is an additional faint band in the upper panel under treatment with PK. This band does not show up in Fig 1D. Do the authors know what is labelled here (e.g. is it a known unspecific band or something different)?

This band was an irregularity of that particular experiment. The experiment was repeated, and this band is no longer visible. The figure has been replaced with the new experiment.

[1.12] The authors switch between American and British English (sometimes even within the same sentence).

We apologise for this and have now changed all to British English.

- The authors are inconsistent in their usage of chemical units: ml vs mL

These have now been double-checked, and all the units are now in mL.

- The authors are inconsistent regarding their use of tenses: e.g. p. 17, line 422-424: "primary antibodies were" vs "secondary antibodies used are"

These have now been fixed.

**Reviewer 2:**

Major points
[2.1] Several mitochondrial TA proteins (like MFF and FIS1) are dually localized to peroxisomes. It will be interesting to test whether Lpg1625 targets also peroxisomes. For example, can it be that the small puncta structures in Fig. 1C, which are not co-stained with the mitochondrial marker, represent peroxisomal structures?

See Reviewer 1 comment [1.2].

[2.2] Fig. 1E: (i) Although possible, the evidence that the charge variants of Lpg1625 are targeted to the ER is not convincing. A co-staining with an ER marker is required.

See Reviewer 1 Comment [1.5].

To support the proposal that the C-tail of Lpg1625 is sufficient for mitochondrial targeting and membrane integration, it will be helpful to show by biochemical means (subcellular fractionation) that this construct is enriched in mitochondrial fraction.

Mitochondria isolated from HeLa cells transfected with ^FLAG^*Lp*PIP^85-130^ (the TA only construct) were subjected to proteinase K treatment and carbonate extraction. The PK treatment shows the protein is degraded in intact mitochondria suggesting an outer membrane localisation. The carbonate extraction shows it is in the pellet fraction, suggesting it is membrane integrated. This allows us to conclude that the TA only construct is localised and embedded in the mitochondrial outer membrane (**Fig S2F**).

[2.3] Fig. 2E: To support the assumption that PPP1CB (or other subunits of PP1) associates with mitochondria upon expression of LpPIP, it will be good to show by western blotting that PP1 is found on isolated mitochondria under these conditions.

See Reviewer 1 comment [1.3]

[2.4] The authors write in lines 221-2 that "This suggests that LpPIP-PP1 holoenzyme could be modulating mitochondria dynamics." To place their finding in a physiological context, the authors should compare mitochondrial morphology in cells harboring LpPIP to cells expressing its FLAG-RVxFdead variant.

We observed no difference in the mitochondrial morphology (length and network) between HeLa cells transfected with ^FLAG^*Lp*PIP or ^FLAG^RVxF_dead_. This claim is now removed from the manuscript (Figs 5C and 5D).

Minor points:
[2.5] Among the four TA proteins, was there a special reason for selecting Lpg1625 as the first candidate for further functional analysis?

We are also investigating the other effectors that we identified in this study. Our initial experiments with Lpg1625 looked most promising and therefore this was the first candidate that we have functionally characterised first.

[2.6] Line 139: As far as I recall, there is no evidence for the involvement of Tom40 in the biogenesis of mitochondrial TA proteins. Thus, the authors might consider rephrasing their text or cite an appropriate reference. Along the same line, if TOM40 is involved in the biogenesis of Lpg1625, it should also interact with the FLAG-RVxFdead variant (Fig. 2D)

We appreciate this insight and have now removed this from the manuscript.

**Line 151:** *Additional co‑immunoprecipitated proteins included MTCH1 and MTCH2 which may facilitate membrane integration of Lpg1625 [4,5]. The VDAC proteins were also co-immunoprecipitated, likely due to their abundance and physical proximity to Lpg1625 in the mitochondrial outer membrane (Fig 2A).*

[2.7] Fig. S2A and S2C: The lanes representing the samples with PK contain a band which migrate slightly slower than FLAG-Lpg1625. The authors should explain this band.
See Reviewer 1 comment [1.11].

[2.8] Line 164: Is Lys16 part of the RVxF motif? The authors should explain this point.
No, but the extra lysine to the N-terminus of RVxF motif can increase affinity for PP1 [6]. This is now clarified and mentioned in the manuscript.

**Line 172:** *Examining the Lpg1625 protein sequence revealed the presence of a RVxF motif within its N‑terminal cytosolic domain (amino acids 17 – 21), with* *two lysine residues upstream of the motif (at position 15 and 16), which can increase affinity for PP1 (Fig 2B) [6].*

[2.9] Lines 245-7: The effect on phosphorylation of nuclear proteins was observed upon overexpression of LpPIP. It will be good if the authors will test this issue also upon lower (and maybe more physiological) expression levels of LpPIP.

We have also conducted whole cell phosphoproteomics using our Flp-In™ T-REx^TM^ 293 stable cell lines which express *Lp*PIP to lower level. This also showed phosphorylation of nuclear proteins, but to a lesser extent. Below is the GO cellular component terms enrichment analysis for the proteins with phosphopeptides that were significantly increased in the stable cell lines expressing ^FLAG^*Lp*PIP compared to ^FLAG^RVxF_dead_. Less terms were enriched compared to Figure S4G and S4H, showing the observation is real, but influenced by the amount of phosphatese, PP1 being recruited away to the mitochondria.

We have not included this data and just add it here to address the comment and for discussion.

[2.10] What is the difference between Fig. 3E and the panel in Fig. S3E. Please explain why they are not identical.
The motif analysis in Figure 3E is based on data from mitochondrial phosphoproteomics (39 phosphosites as input), and Figure S3E is based on whole cell phosphoproteomics (115 phosphosites as input). The difference in sample size causes the difference, but the significant enrichment of arginine at position -3 remains the same between the two. This is now clarified in the figure legends for Figure 3E and S3E.

**Reviewer 3:**
[3.1] Mitochondrial morphology of transiently expressed effector proteins Lpg1803 and Lpg2344 do not appear to overlap exclusively with NDUFAF2, suggesting either non-mitochondrial localization, or disruption to mitochondrial integrity. Expression of Lpg1625 (LpPIP), Lpg1803, and Lpg2344 appears to impact overall mitochondrial morphology. Given the identification of mitochondrial dynamics as an enriched GO term for phosphoproteins regulated in response to LpPIP expression, the authors could examine if there are quantifiable differences in morphology between wild type, LpPIP and RVxF dead cells. Additionally, higher magnification images should be shown to support the conclusions drawn in the manuscript.
Lpg2344 was indeed found to also localise to the cytosol in our studies, but a GFP-tagged version of this protein has been reported by others to be mitochondrial. Lpg1803, however, shows mitochondrial localisation and does not disrupt mitochondrial integrity based on our analysis. The image is now changed in Fig 1C to avoid confusion.

Mitochondrial morphology (length and network) has been assessed as outlined above is not influenced by *Lp*PIP (Figs 4D and 5D).

[3.2] The authors should provide representative images that were used in generating the results shown in Fig. 4B.

The representative images are now added as S4B Fig.

[3.3] The changes in mitochondrial protein phosphorylation suggest altered functionality of, for example, protein import. Is there a measurable effect on this or any other process related to the phosphoproteins?

Please see responses to Editor and Reviewer 1. We specifically looked at carrier protein import to address this comment by reviewer 3, but import of Tim23 and GC1, two TIM22 substrates that use the Tomm70 receptor were not influenced by the presence of *Lp*PIP.
