# Supplementary material for: Legionella effector LpPIP recruits protein phosphatase 1 to the mitochondria to induce dephosphorylation of outer membrane proteins
Source: PLoS Biol. 2025 Jul 23;23(7):e3003261. doi: 10.1371/journal.pbio.3003261 (PMC12313075; doi:10.1371/journal.pbio.3003261)
Supplement: Attachment — Submitted filename: PLOS Biol_revision 2 Response Letter.docx [file pbio.3003261.s022.docx]

**PBIOLOGY-D-24-03153R2** – Response Letter Revision 2

**Reviewer 1** and **Reviewer 3** have recommended puclication and are satisfied with the new data and revisions.

**Reviewer 2:**
The reviewer has raised two minor points, these are addressed below.

[1] Fig. S2F: The PK treatment is not mentioned in the text and is also not convincing as the inner membrane protein TIM29 is also affected. I would suggest removing this part from the figure.

We agree with the reviewer and have removed the PK treatment. The important part of this Figure was the carbonate extraction, which still remains and updated as S2 Fig. This is also reflected in the text – line 169: We confirmed FLAGLpg162585-130 (TMD and C-tail) was also sufficient for membrane integration using carbonate extraction (S2F Fig).

[2] Lines 154-156 and Fig. S3: the authors write: "To rule out potential artifacts, we generated a stable tetracycline-inducible Flp-InTMT-RExTM 293 cell line expressing FLAGLpg1625, allowing regulation of expression to low levels." However, they do not show that this cell line indeed expresses less FLAGLpg1625 as compared to the cells used in the previous assays (Figs. 1 and 2). A comparison by Western blot where both cell lines are analysed side-by-side will be helpful.

This has been completed and is shown below for the purpose of this revision. We have not included this in the manuscript. The gel below shows the stable cells (15-120 ug if mitochondrial protein) in lanes 1-5 and the transiently expressed protein (15-60 ug of protein) in lanes 8-10. You can see the transient system expresses significantly more *Lp*PIP with detection visible at 15 ug.

**Editors requests covered in email:**

[1] In addition, we would like you to consider a suggestion to improve the title:
"**Legionella effector LpPIP recruits protein phosphatase 1 to the mitochondria to induce dephosphorylation of outer membrane proteins**"

Thank you for this recommendation. This change has been made.

[2] Review your reference list to ensure that it is complete and correct. If you have cited papers that have been retracted, please include the rationale for doing so in the manuscript text or remove these references and replace them with relevant current references. Any changes to the reference list should be mentioned in the cover letter that accompanies your revised manuscript.

The following changes have been made:

[2.1] **Added reference for PRIDE**:

Reference 96. Perez-Riverol Y, Bai J, Bandla C, García-Seisdedos D, Hewapathirana S, Kamatchinathan S, et al. The PRIDE database resources in 2022: a hub for mass spectrometry-based proteomics evidences. Nucleic Acids Research. 2022;50: D543–D552. doi:10.1093/nar/gkab1038

[2.2] **Reference 20.** Escoll P, Platon L, Dramé M, Sahr T, Schmidt S, Rusniok C, et al. Reverting the mode of action of the mitochondrial FOF1-ATPase by Legionella pneumophila preserves its replication niche. ~~Blokesch M, Storz G, editors.~~ eLife. 2021;10: e71978. doi:10.7554/eLife.71978

[2.3] **Reference 23**. Fu J, Zhou M, Gritsenko MA, Nakayasu ES, Song L, Luo Z-Q. *Legionella pneumophila* modulates host energy metabolism by ADP-ribosylation of ADP/ATP translocases. ~~Helaine S, Soldati-Favre D, Newton H, Hartland E, editors.~~ eLife. 2022;11: e73611. doi:10.7554/eLife.73611

[2.4] **Update Reference 80 from preprint to the published article:**

**Preprint:** 80. Wei L, Gok MO, Svoboda JD, Forny M, Friedman JR, Niemi NM. PPTC7 limits mitophagy through proximal and dynamic interactions with BNIP3 and NIX. bioRxiv. 2024; 2024.01.24.576953. doi:10.1101/2024.01.24.576953

To:

**Accepted manuscript:** 80. Wei L, Gok MO, Svoboda JD, Kozul K-L, Forny M, Friedman JR, et al. Dual-localized PPTC7 limits mitophagy through proximal and dynamic interactions with BNIP3 and NIX. Life Science Alliance. 2024;7. doi:10.26508/lsa.202402765

[2.5] Updated reference for 88 to include Editors for the Book chapter:

Previous reference 88. Korevaar E, Khoo CA, Newton HJ. Genetic Manipulation of Non-pneumophila Legionella: Protocols Developed for Legionella longbeachae. Methods Mol Biol. 2019;1921: 145–157. doi:10.1007/978-1-4939-9048-1_9

To:

New reference 88. Korevaar E, Khoo CA, Newton HJ. Genetic Manipulation of Non-*pneumophila Legionella*: Protocols Developed for *Legionella longbeachae*. In: Buchrieser C, Hilbi H, editors. Legionella: Methods and Protocols. New York, NY: Springer; 2019. pp. 145–157. doi:10.1007/978-1-4939-9048-1_9

[3] You may be aware of the PLOS Data Policy, which requires that all data be made available without restriction: <http://journals.plos.org/plosbiology/s/data-availability>. For more information, please also see this editorial: <http://dx.doi.org/10.1371/journal.pbio.1001797>

I can see that you have some of the data on the tables, however we do require **all individual quantitative observations** that underlie the data summarized in the graphs shown in the figures and results of your paper be made available in one of the following forms:
1) **Supplementary files** or 2) **Deposition in a publicly available repository**.

Fig. 2A, D, E, F, G; Fig. 3B-D, F; Fig. 4A, B, D, E, G, H, I, J; Fig. 5A-F; Fig. S1; Fig. S2B-D; Fig. S3B, D and Fig. S4A, C, D, G, H

We have included all data requested as Supp Files. These include S1_Data to S4_Data. These data sets have been references in the relevant Figure legends.

[4] Please ensure that your **Data Statement in the submission system** accurately describes where your data can be found.

Done

[5] ***In addition, please make the data you have deposited in the ProteomeXchange Consortium via the PRIDE (PXD057005) publicly available at this stage.

This is now publicly available and indicated in the text on Line 1004: The mass spectrometry proteomics data have been deposited to the ProteomeXchange Consortium via the PRIDE [98] partner repository with the dataset identifier PXD057005.

**Journal Requirements:**

[6] Your current Financial Disclosure states, “ This work was funded by the Australian Research Council (DP240101332) to DS and HN. ”.

However, your **funding information on the submission form** indicates that you did not receive funding. Please indicate by return email the full and correct funding information for your study and confirm the order in which funding contributions should appear. Please be sure to indicate whether the funders played any role in the study design, data collection and analysis, decision to publish, or preparation of the manuscript.

We apologise for this. The correct information is the information indicated above: This work was funded by the Australian Research Council (DP240101332) to DS and HN. The funders have played no part in study design, data collection or analaysis, or decisions to publish or in the preparation of this manuscript.

[7] Please amend your **detailed Financial Disclosure statement**. This is published with the article, therefore should be completed in full sentences and contain the exact wording you wish to be published.
i). State what role the funders took in the study. If the funders had no role in your study, please state: “collection and analysis, decision to publish, or preparation of the manuscript.”
ii). If any authors received a salary from any of your funders, please state which authors and which funders.

This has been addressed in the Financial Disclosure Statement.

[8] Please note that we cannot publish copyright symbols such as ©, ®, or ™. We are also unable to publish logos or other brand-related content.

These changes have been made in text and changes tracked.

[9] "Please refer to the following article for PLOS Biology's best-practice recommendations for data presentation: <https://journals.plos.org/plosbiology/article?id=10.1371/journal.pbio.1002128>.
We have reviewed this and are following all of your guidelines to the best of our knowledge.
